# Identifications of novel host cell factors that interact with the receptor-binding domain of the SARS-CoV-2 spike protein

**DOI:** 10.1016/j.jbc.2024.107390

**Published:** 2024-05-21

**Authors:** Xiao Tang, Yang Liu, Jinhui Wang, Teng Long, Bobo Wing Yee Mok, Yan Huang, Ziqing Peng, Qinyu Jia, Chengxi Liu, Pui-Kin So, Sirius Pui-Kam Tse, Cheuk Hei NG, Shiyi Liu, Fei Sun, Shaojun Tang, Zhong-Ping Yao, Honglin Chen, Yusong Guo

**Affiliations:** 1 Anhui Provincial Key Laboratory of Molecular Enzymology and Mechanism of Major Metabolic Diseases, College of Life Sciences, Anhui Normal University, Wuhu, China; 2 Division of Life Science and State Key Laboratory of Molecular Neuroscience, The Hong Kong University of Science and Technology, Hong Kong SAR, China; 3 Department of Microbiology, The University of Hong Kong, Hong Kong SAR, China; 4 Centre for Virology, Vaccinology and Therapeutics Limited, The University of Hong Kong, Pokfulam, Hong Kong SAR, China; 5 State Key Laboratory of Chemical Biology and Drug Discovery, Research Institute for Future Food, Research Centre for Chinese Medicine Innovation, and Department of Applied Biology and Chemical Technology, The Hong Kong Polytechnic University, Kowloon, Hong Kong SAR, China; 6 Thrust of Bioscience and Biomedical Engineering, Hong Kong University of Science and Technology (Guangzhou), Guangzhou, China; 7 Department of Chemical and Biological Engineering, Hong Kong University of Science and Technology, Hong Kong SAR, China; 8 Hong Kong University of Science and Technology, Shenzhen Research Institute, Shenzhen, China

**Keywords:** SARS-CoV-2, the receptor binding domain of spike, viral entry, ACE2, SH3BP4, integrin

## Abstract

SARS-CoV-2 entry into host cells is facilitated by the interaction between the receptor-binding domain of its spike protein (CoV2-RBD) and host cell receptor, ACE2, promoting viral membrane fusion. The virus also uses endocytic pathways for entry, but the mediating host factors remain largely unknown. It is also unknown whether mutations in the RBD of SARS-CoV-2 variants promote interactions with additional host factors to promote viral entry. Here, we used the GST pull-down approach to identify novel surface-located host factors that bind to CoV2-RBD. One of these factors, SH3BP4, regulates internalization of CoV2-RBD in an ACE2-independent but integrin- and clathrin-dependent manner and mediates SARS-CoV-2 pseudovirus entry, suggesting that SH3BP4 promotes viral entry *via* the endocytic route. Many of the identified factors, including SH3BP4, ADAM9, and TMEM2, show stronger affinity to CoV2-RBD than to RBD of the less infective SARS-CoV, suggesting SARS-CoV-2–specific utilization. We also found factors preferentially binding to the RBD of the SARS-CoV-2 Delta variant, potentially enhancing its entry. These data identify the repertoire of host cell surface factors that function in the events leading to the entry of SARS-CoV-2.

Since 2020, the rapid global spread of the highly pathogenic SARS-CoV-2 virus has led to a huge public health crisis. The SARS-CoV-2 genome shares approximately 80% identity with SARS-CoV, the virus responsible for the 2003 epidemic (1). Both these viruses are characterized by a densely glycosylated spike (S) protein that heavily coat their surface. The S protein is a trimeric protein composed of two subunits, S1 and S2. A subdomain of S1, known as the receptor-binding domain (CoV2-RBD), directly binds to the cell surface receptor angiotensin-converting enzyme 2 (ACE2) (1, 2, 3, 4). This interaction plays a critical role in viral attachment. Following viral attachment, the S2 subunit undergoes cleavage by the host cell-surface–located protease TMPRSS2. This process releases the fusion peptide, initiating fusion between the viral and host membranes. Subsequently, the viral genome is released into the cytoplasm of host cell, marking the next stage in the viral life cycle (5). In conditions where the spike protein does not engage with TMRSS2, SARS-CoV-2 can enter the host cells by the endocytic pathway (6). In this scenario, the S2 subunit is cleaved by cathepsins in the acidic environment of the endosomes, activating fusion between the viral and endosome membrane (6). However, the specific host factors that drive this endocytic process remain largely undefined.

CoV2-RBD interacts with ACE2 with a stronger affinity than its SARS-CoV counterpart, CoV-RBD (7, 8), thereby enhancing the infectivity of SARS-CoV-2. Whether other host factors show a stronger affinity for CoV2-RBD over CoV-RBD, potentially escalating the viral infection, remains to be further investigated. The CoV2-RBD, besides its role in binding to host receptors, also functions as the primary target for antibodies in individuals infected with SARS-CoV-2. Most mutations in SARS-CoV2 variants are observed within this RBD region, which allows the virus to escape immune surveillance. However, it is still unclear whether these mutations permit RBD the ability to interact with other host factors, which could potentially enhance the efficiency of viral entry.

To better understand the molecular mechanisms regulating the viral entry, it is crucial to identify the repertoire of host cell surface factors that interact with CoV2-RBD that facilitate viral infection. While proteomic studies have discovered numerous interactors of the S protein (9, 10), many previous studies have not detected interactions between the S protein and ACE2. One potential explanation is the low expression levels of ACE2 in the experimental cell lines used in these studies. Another possibility is that the full-length S protein employed in the research has reduced affinity to ACE2 due to a significant proportion of the RBD in the full-length S protein adopting a closed conformation, which renders the ACE2-binding site on the RBD inaccessible (2, 7).

In this study, we investigated the interaction between CoV2-RBD and host cell factors by using purified GST-tagged CoV2-RBD as a bait. We examined the functional roles of key amino acids in CoV2-RBD that mediate CoV2-RBD-ACE2 interaction. Furthermore, we identified novel host factors that bind to CoV2-RBD. One such factor, SH3 domain-binding protein 4 (SH3BP4), regulates the internalization of CoV2-RBD in HeLa cells by an ACE2-independent but integrin- and clathrin-dependent manner. Further analysis indicates that SH3BP4 mediates the entry of lentivirus pseudotyped with the parental, Delta, or Omicron BA2 variant of the SARS-CoV-2 spike. Interestingly, many of the discovered host factors display enhanced binding affinity to CoV2-RBD compared to CoV-RBD, suggesting that these host factors may be specifically utilized by SARS-CoV-2 to facilitate viral entry. We also uncovered surface-located host factors that preferentially bind to RBD of the SARS-CoV-2 Delta variant compared to the prototype, indicating that these factors may promote the entry of SARS-CoV-2 Delta variant. Our data reveal novel host factors interacting with RBD and provide insights into roles of the RBD of S protein in viral entry.

## Results

### Reconstitution of the interaction between CoV2-RBD and ACE2 through GST pull-down assay

**Figure 1:**
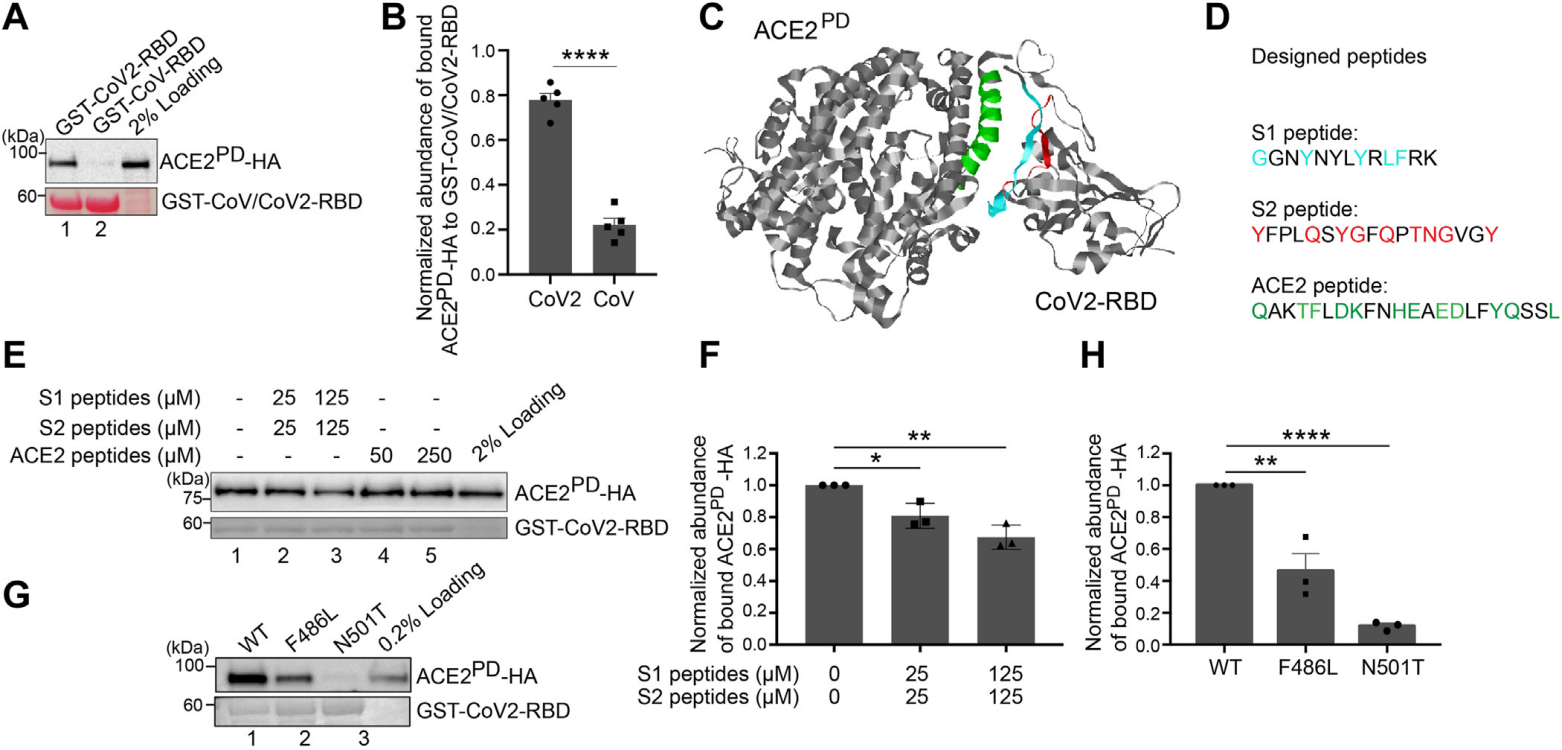
**Reconstitution of the interaction between CoV2-RBD and ACE2**^**PD**^**through GST pull-down.***A*, purified GST, GST-tagged CoV-RBD, or GST-tagged CoV2-RBD was incubated with lysates from HEK293T cells transfected with ACE2^PD^-HA. After incubation, the bound proteins were analyzed by immunoblotting with anti-HA antibodies. *B*, relative abundances of ACE2^PD^-HA that bound to GST-CoV2-RBD or GST-CoV-RBD were quantified (n = 5, mean ± S.D.). In each biological repeat, the abundance of ACE2^PD^-HA that bound to GST-CoV2-RBD or GST-CoV-RBD was normalized to the corresponding bait protein, and this value was then normalized to the sum of the normalized abundance of bound ACE2^PD^-HA in all experimental groups. ∗∗∗∗*p* < 0.0001. *C*, the structure of CoV2-RBD-ACE2 complex (PDB ID: 6M0J). S1 peptide, S2 peptide, and ACE2 peptide are highlighted in *cyan*, *red*, and *green*, respectively. *D*, the amino acid sequence of designed peptides. *E*, purified GST-tagged CoV2-RBD was incubated with lysates from HEK293T cells transfected with ACE2^PD^-HA in the presence of the indicated concentrations of S1, S2, or ACE2 peptides. After incubation, the bound proteins were analyzed by immunoblotting with anti-HA antibodies. *F*, relative abundances of ACE2^PD^-HA that bound to GST-CoV2-RBD were quantified (n = 3, mean ± S.D.). The quantification is normalized to the abundance of ACE2^PD^-HA that bound to GST-CoV2-RBD in the absence of peptides in each experimental group. ∗*p* < 0.05; ∗∗*p* < 0.01. *G*, purified GST-tagged WT CoV2-RBD or the indicated mutant variant of CoV2-RBD was incubated with lysates from HEK293T cells transfected with ACE2^PD^-HA. After incubation, the bound proteins were analyzed by immunoblotting with anti-HA antibodies. *H*, relative abundances of ACE2^PD^-HA that bound to GST-CoV2-RBD, GST-CoV2-RBD^F486L^, or GST-CoV2-RBD^N501T^ were quantified (n = 3, mean ± S.D.). The quantification is normalized to the abundance of ACE2^PD^-HA that bound to WT GST-CoV2-RBD in each experimental group. ∗∗*p* < 0.01; ∗∗∗∗*p* < 0.0001. ACE2, angiotensin-converting enzyme 2.

The extracellular domain of ACE2 contains a peptidase domain (ACE2^PD^) that directly interacts with CoV2-RBD (11). We performed GST pull-down experiments to reconstitute the interaction between CoV2-RBD and ACE2^PD^. Purified GST-tagged spike RBD from SARS-CoV (GST-CoV-RBD) or SARS-CoV-2 (GST-CoV2-RBD) were incubated with cell lysates from HEK293T cells expressing HA-tagged ACE2^PD^ (ACE2^PD^-HA) and then the bound proteins were analyzed by Western blot. We found that the abundance of ACE2^PD^-HA that bound to GST-CoV2-RBD was significantly higher than that bound to GST-CoV-RBD (Fig. 1*A* and quantification in Fig. 1*B*). This analysis suggests that CoV2-RBD has a stronger binding efficiency with ACE2^PD^ than CoV-RBD, which is consistent with previous reports (7, 8).

The structure of CoV2-RBD in association with ACE2^PD^ has been determined (8, 12). Seventeen residues of receptor-binding motif on CoV2-RBD have been revealed to directly interact with 20 residues of ACE2^PD^ (8). Eleven of the twenty residues of ACE2 are located at the N-terminal helix of ACE2 (8). Based on these structural analyses, we synthesized three experimental peptides: (1) a peptide corresponding to the amino acid sequence 446 to 458 of CoV2-RBD (Figs. 1*C*, S1 peptide, highlighted in cyan), (2) a peptide corresponding to the amino acid sequence 489 to 505 of CoV2-RBD (Figs. 1*C*, S2 peptide, highlighted in red), and (3) a peptide corresponding to the N-terminal helix of ACE2 (amino acid sequence 24–45) (Fig. 1*C*, ACE2 peptide, highlighted in green). Based on structural analyses (8, 12), these peptides contain residues that directly interact with ACE2 or CoV2-RBD (Fig. 1*D*, highlighted in cyan, red or green). Peptides partially reduced the interaction between ACE2^PD^-HA and CoV2-RBD when they were used in combination (Fig. 1*E* and quantification in Fig. 1*F*). These results are consistent with the structural analysis, suggesting that the binding sites on CoV2-RBD predicted by the structural analysis indeed contribute to the interaction between CoV2-RBD and ACE2^PD^. However, peptides corresponding to the N-terminal helix of ACE2 (ACE2 peptides) did not interfere with this interaction (Fig. 1*E*). As residues outside of the N-terminal helix of ACE2 also contribute to the ACE2-RBD interaction (8), ACE2 peptides may not be competent to induce the releases of ACE2^PD^ from CoV2-RBD.

Structural analysis of CoV-RBD-ACE2^PD^ and CoV2-RBD-ACE2^PD^ and sequence alignment of CoV-RBD and CoV2-RBD revealed 14 shared positions of residues that are involved in the interaction. Among these positions, the residues at five positions are different between CoV-RBD and CoV2-RBD (8). To test whether these positions are important for RBD-ACE2 interaction, we substituted two of them, F486 and N501, in CoV2-RBD to the corresponding residues in CoV-RBD, L472, and T487, respectively (F486L and N501T). The abundance of ACE2^PD^-HA that bound to mutant versions of GST-CoV2-RBD was significantly lower than that bound to wild-type GST-CoV2-RBD (Fig. 1*G* and quantification in 1*H*), demonstrating that the changes of residues on these positions enhance the interaction between CoV2-RBD and its receptor ACE2.

### Analyses of the interaction between CoV2-RBD variants and ACE2

**Figure 2:**
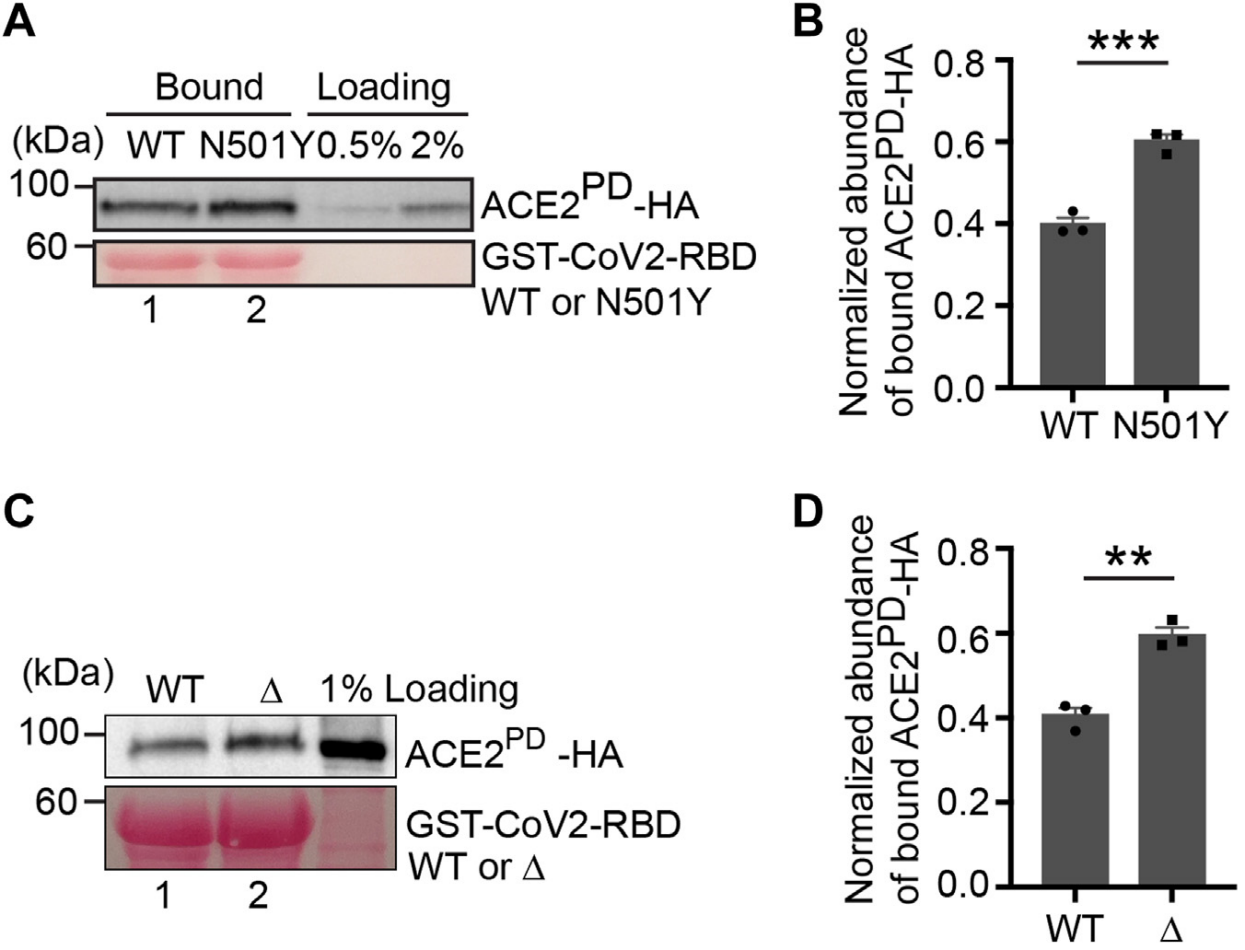
**Analyses of the interaction between CoV2-RBD variants and ACE2.***A*, purified GST-tagged WT CoV2-RBD or the variant of CoV2-RBD, N501Y, was incubated with lysates from HEK293T cells transfected with ACE2^PD^-HA. After incubation, the bound proteins were analyzed by immunoblotting with anti-HA antibodies. *B*, relative abundances of ACE2^PD^-HA that bound to GST-CoV2-RBD or GST-CoV2-RBD^N501Y^ were quantified (n = 3, mean ± S.D.). ∗∗∗*p* < 0.0001. *C*, purified GST-tagged WT CoV2-RBD or the Delta variant (CoV2-RBD^Δ^), was incubated with lysates from HEK293T cells transfected with ACE2^PD^-HA. After incubation, the bound proteins were analyzed by immunoblotting with anti-HA antibodies. *D*, relative abundances of ACE2^PD^-HA that bound to GST-CoV2-RBD or GST-CoV2-RBD^Δ^ were quantified (n = 3, mean ± S.D.). ∗∗*p* < 0.01. In each biological repeat, the abundance of bound ACE2^PD^-HA was normalized to the corresponding bait protein, and this value was then normalized to the sum of the normalized abundance of bound protein in all experimental groups (*B* and *D*). ACE2, angiotensin-converting enzyme 2.

Five variants of SARS-CoV-2, including Alpha, Beta, Gamma, Delta, and Omicron, have been classified as variants of concern by the World Health Organization. Genomic sequencing results revealed that N501Y on spike-RBD is the common mutation of Alpha, Beta, and Gamma (13). The Delta variant contains L452R and T478K substitutions on CoV2-RBD. Controversial evidence is reported to show whether these mutations in the Delta variant enhance the ACE2–spike interaction (14, 15, 16). We performed GST pull-down approach to explore the roles of key residues mutated in CoV2-RBD variant in binding to ACE2. We found that the abundance of ACE2^PD^ that bound to GST-CoV2-RBD bearing N501Y substitution or Delta variant (GST-CoV2-RBD^N501Y^ or GST-CoV2-RBD^Δ^) was significantly higher than that bound to GST-CoV2-RBD (Fig. 2, *A* and *C*, compare lanes 1 and 2, and quantification in 2, *B* and *D*). These analyses indicate that the N501Y mutation and the mutations in the Delta variant enhanced the interaction between RBD and ACE2^PD^. These results and previous reports (15, 16, 17, 18, 19) provide an explanation for the stronger transmissibility of these variants.

### The integrin-binding motif (RGD) in CoV2-RBD is important for CoV2-RBD to bind to integrin β3 and ACE2^PD^

**Figure 3:**
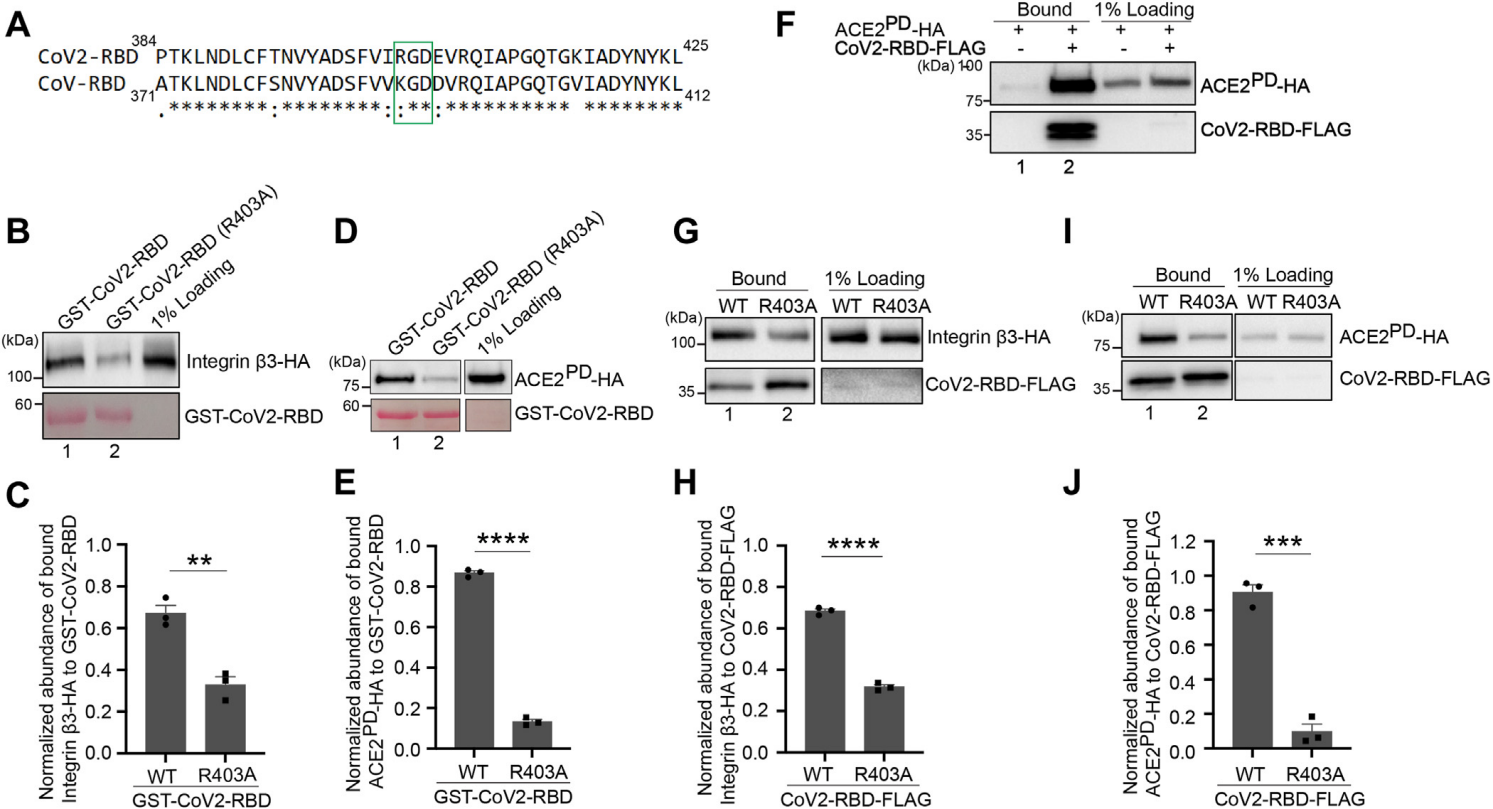
**The RGD motif in CoV2-RBD is important for CoV2-RBD to bind to ACE2**^**PD**^**and integrin.***A*, sequence alignment of CoV2-RBD and CoV-RBD. *B*, purified GST-tagged WT CoV2-RBD or CoV2-RBD^R403A^ was incubated with lysates from HEK293T cells transfected with integrin β3-HA. After incubation, the bound proteins were analyzed by immunoblotting with anti-HA antibodies. *C*, relative abundances of integrin β3-HA that bound to GST-CoV2-RBD or GST-CoV2-RBD^R403A^ were quantified (n = 3, mean ± S.D.). ∗∗*p* < 0.01. *D*, purified GST-tagged WT CoV2-RBD or CoV2-RBD^R403A^ was incubated with lysates from HEK293T cells transfected with ACE2^PD^-HA. After incubation, the bound proteins were analyzed by immunoblotting with anti-HA antibodies. *E*, relative abundances of integrin β3-HA that bound to GST-CoV2-RBD or GST-CoV2-RBD^R403A^ were quantified (n = 3, mean ± S.D.). ∗∗∗∗*p* < 0.0001. *F*, lysates from HEK293T cells transfected with plasmids encoding the indicated constructs were incubated with anti-FLAG affinity gel. The bound proteins were eluted by FLAG peptides, and the eluted fractions were analyzed by immunoblotting. *G*, lysates from HEK293T cells cotransfected with plasmids encoding integrin β3-HA and WT CoV2-RBD-FLAG or CoV2-RBD^R403A^-FLAG were incubated with anti-FLAG affinity gel. The bound proteins were eluted by FLAG peptides, and the eluted fractions were analyzed by immunoblotting. *H*, relative abundances of integrin β3-HA that bound to CoV2-RBD-FLAG or CoV2-RBD^R403A^-FLAG were quantified (n = 3, mean ± S.D.). ∗∗∗∗*p* < 0.0001. *I*, lysates from HEK293T cells cotransfected with plasmids encoding ACE2^PD^-HA and WT CoV2-RBD-FLAG or CoV2-RBD^R403A^-FLAG were incubated with anti-FLAG affinity gel. The bound proteins were eluted by FLAG peptides, and the eluted fractions were analyzed by immunoblotting. *J*, relative abundances of ACE2^PD^-HA that bound to CoV2-RBD-FLAG or CoV2-RBD^R403A^-FLAG were quantified (n = 3, mean ± S.D.). ∗∗∗*p* < 0.001. In each biological repeat, the abundance of bound protein was normalized to the corresponding bait protein, and this value was then normalized to the sum of the normalized abundance of bound protein in all experimental groups (*C*, *E*, *H*, and *J*). ACE2, angiotensin-converting enzyme 2.

We then tested the interactions between CoV2-RBD and other host factors. CoV2-RBD contains RGD, a classical integrin-binding motif (20). The RGD motif lies close to the region of CoV2-RBD specifically involved in binding to ACE2^PD^ (21). Sequence alignment indicates that CoV-RBD also contains a similar motif, KGD, at the corresponding position (Fig. 3*A*). The RGD motif in S protein is important for its interaction with multiple integrin subtypes, such as integrin αvβ3, integrin αvβ5, and integrin α5β1 heterodimer (22, 23, 24, 25). We found that purified GST-CoV2-RBD interacted with HA-tagged integrin β3 (integrin β3-HA) from cell lysates (Fig. 3*B*, lane 1). In contrast, mutating the RGD motif to AGD severely blocked the interaction between GST-CoV2-RBD and integrin β3-HA (Fig. 3*B* and quantification in 3*C*). Integrin β3 is reported to bind with various extracellular matrix proteins and regulate cell migration and cell adhesion (26, 27, 28). Our study indicates that this protein is also a binding partner of CoV2-RBD. Interestingly, mutating the RGD motif to AGD also blocked the interaction between GST-CoV2-RBD and ACE2^PD^-HA (Fig. 3*D* and quantification in 3*E*), suggesting this motif is also important for ACE2 to interact with the RBD of S.

As an additional test, we performed coimmunoprecipitation using HEK293T cells co-expressing FLAG-tagged human codon–optimized CoV2-RBD (CoV2-RBD-FLAG) (29) with integrin β3-HA or ACE2^PD^-HA. The results confirmed the interaction between ACE2^PD^ and CoV2-RBD (Fig. 3*F*). Consistent with the results of GST pull-down, coimmunoprecipitation analysis indicates that CoV2-RBD-FLAG interacted with integrin β3-HA and ACE2^PD^, and mutating the RGD motif significantly reduced these interactions (Fig. 3, *G*–*J*). These analyses are consistent with a previous report demonstrating that the arginine residue at the position 403 of S is important for binding of S to ACE2 (30).

### GST-CoV2-RBD is efficiently internalized in HeLa cells and this process is clathrin- and RGD-motif–dependent but is independent of ACE2

After viral attachment, SARS-CoV2 can be delivered to the endosomes of the host cells through the endocytic pathway (5). However, the molecular mechanism regulating this step remains largely unclear. Purified full-length SARS-CoV2 spike proteins are shown to be internalized into mammalian cells in an ACE2-dependent manner through clathrin-mediated endocytosis (31). CoV2-RBD–tagged extracellular vesicles are shown to be internalized into mammalian cell lines or organoids which have high levels of endogenous ACE2, including Caco-2 cells, human iPS cells-derived cardiomyocytes, and intestinal organoids (32). Purified His-tagged CoV2-RBD are shown to be internalized into AGS cells which ectopically express ACE2 (33).

**Figure 4:**
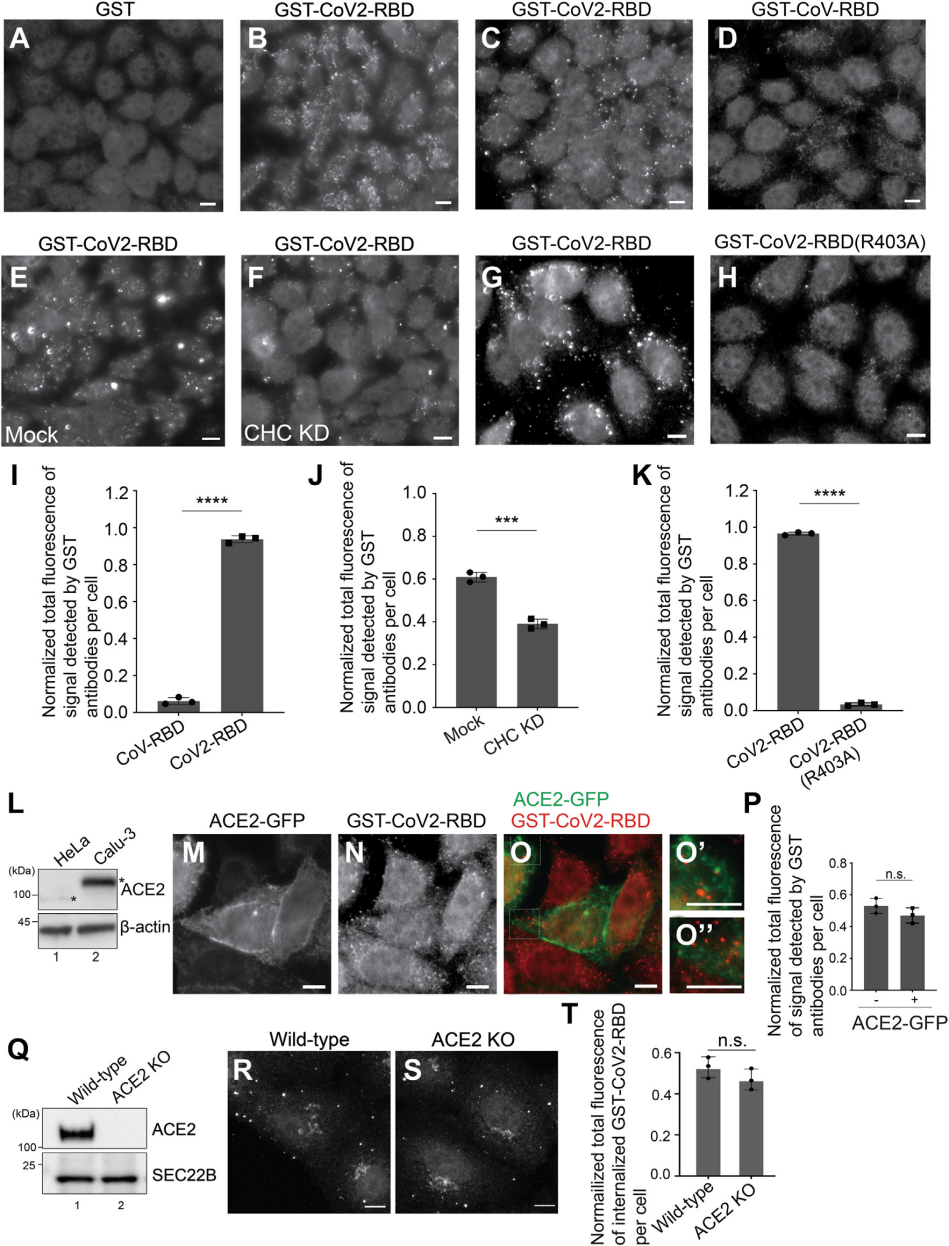
**CoV2-RBD can be efficiently internalized in HeLa cells in a clathrin- and RGD-motif–dependent but ACE2-independent manner.***A–D*, *G* and *H*, 5 ng/μl purified GST (*A*), GST-CoV2-RBD (*B*, *C*, and *G*), GST-CoV-RBD (*D*), or GST-CoV2-RBD^R403A^ (*H*) were incubated with HeLa cells at 37 °C. Thirty minutes after incubation, the presence of the purified protein was detected by immunofluorescence. The scale bar represents 10 μm. *E* and *F*, HeLa cells were transfected with NC siRNA (*E*) or siRNA against CHC (*F*). 48 h after transfection, 5 ng/μl purified GST-CoV2-RBD was incubated with HeLa cells at 37 °C for 30 min. The presence of the purified protein was detected by immunofluorescence. The scale bar represents 10 μm. *I*–*K*, quantifications of the total fluorescence of the signal in each cell (n = 3, mean ± S.D., over 100 cells were quantified in each experimental group). The level of the total fluorescence in each group was normalized to the sum of the level of fluorescence in two experimental groups. ∗∗∗*p* < 0.001; ∗∗∗∗*p* < 0.0001. *L*, the level of endogenous ACE2 and β-actin in cell lysates from HeLa cells or Calu-3 cells were analyzed by immunoblotting with antibodies against ACE2 and β-actin. *Asterisks* indicate the position of ACE2 in HeLa cells or Calu-3 cells. *M*–*O*, HeLa cells were transfected with plasmids encoding GFP-tagged ACE2. Twenty four hours after transfection, 5 ng/μl purified GST-CoV2-RBD was incubated with HeLa cells at 37 °C for 30 min. The presence of the purified protein was detected by immunofluorescence. The scale bar represents 10 μm. The magnified views of the indicated area in panels *O* were shown in panels *O′* and *O’’*. The scale bar represents 10 μm. *P*, quantifications of the total fluorescence of the signal in each cell (n = 3, mean ± S.D., over 100 cells were quantified in each experimental group). In each biological repeat, the level of the total fluorescence in each group was normalized to the sum of fluorescent level in all experimental groups. n.s., not significant. *Q*, the level of endogenous ACE2 and SEC22B in cell lysates from WT or ACE2 KO Vero E6 cells were analyzed by immunoblotting with antibodies against ACE2 and SEC22B. *R* and *S*, purified GST-CoV2-RBD was incubated with WT or ACE2 KO Vero E6 cells at 37 °C for 30 min. The presence of the internalized protein was detected by immunofluorescence. The scale bar represents 10 μm. *T*, quantifications of the total fluorescence of the internalized GST-CoV2-RBD signal in each cell (n = 3, mean ± S.D., over 100 cells were quantified in each experimental group). In each biological repeat, the level of the total fluorescence in each group was normalized to the sum of fluorescent level in all experimental groups. ACE2, angiotensin-converting enzyme 2; CHC, clathrin heavy chain; n.s., not significant.

In this study, we analyzed the internalization of the purified GST-tagged RBD of S. We found that purified GST-CoV2-RBD was efficiently internalized into HeLa cells, whereas purified GST was not (Fig. 4, *A* and *B*). Many of the internalized GST-CoV2-RBD punctate structures were colocalized with EGFR (Fig. S1, *A*–*K*). The internalization efficiency of GST-CoV2-RBD was significantly higher than that of GST-CoV-RBD (Fig. 4, *C* and *D*, and quantification in 4*I*). To test whether clathrin mediates the internalization of GST-CoV2-RBD in HeLa cells, we transfected HeLa cells with siRNA against clathrin heavy chain (CHC) to reduce the expression of CHC (34). We found that the internalization of GST-CoV2-RBD was partially blocked in the CHC knockdown cells (Fig. 4, *E* and *F*, and quantification in 4*J*), suggesting that clathrin regulates the internalization of GST-CoV2-RBD into HeLa cells. In addition, we found that mutating the RGD motif to AGD severely reduced the efficiency of internalization (Fig. 4, *G* and *H*, and quantification in 4*K*) which is consistent with a previous report (24). These analyses suggest that CoV2-RBD is internalized in HeLa cells in a clathrin-dependent and RGD-motif–dependent manner.

GST-CoV-RBD and GST-CoV2-RBD^R403A^ showed lower binding efficiency with ACE2^PD^ than GST-CoV2-RBD (Figs. 1, *A* and *B* and 3, *D* and *E*). We found that the efficiency of internalization of GST-CoV-RBD and GST-CoV2-RBD^R403A^ was also greatly reduced, indicating that ACE2 may regulate the internalization of CoV2-RBD. However, Western blot analysis indicates that endogenous ACE2 proteins in HeLa cell lysates were marginally detected by ACE2 antibodies, whereas endogenous ACE2 proteins in lung epithelial cells (Calu-3) were clearly detected (Fig. 4*L*, asterisks indicate the position of ACE2). The sizes of ACE2 in these two cell lines are different, probably due to different posttranslational modifications. To explore whether ACE2 is important in CoV2-RBD internalization in HeLa cells, we incubated GST-CoV2-RBD with HeLa cells transiently transfected with plasmids encoding GFP-tagged ACE2 (ACE2-GFP). Unexpectedly, the efficiency of GST-CoV2-RBD internalization in HeLa cells not expressing ACE2-GFP is comparable with that in HeLa cells expressing ACE2-GFP (Fig. 4, *M*–O, quantification in 4*P*). ACE2-GFP showed a cell surface localization pattern in the majority of cells but not co-internalized with GST-CoV2-RBD (Fig. 4, *M*–*O*). ACE2-GFP located to punctate structures in some of the expressing cells, and the internalized RBD was not colocalized with ACE2 puncta (Fig. 4*O*, magnified views in Fig. 4, *O*’-O’’). Since HeLa cells express a low level of ACE2, we knockout ACE2 in Vero E6 cells using CRISPR-Cas9 (Fig. 4*Q*). We incubated GST-CoV2-RBD with WT Vero E6 cells or ACE2 knockout Vero E6 cells. We found that the efficiency of GST-CoV2-RBD internalization in ACE2 KO Vero E6 cells is similar to that observed in WT Vero E6 cells (Fig. 4, *R* and *S*, quantification in 4T). This analysis suggests that ACE2 is not important for the internalization of CoV2-RBD in Vero E6 cells.

### Identification of additional host cell factors that preferentially bind to GST-CoV2-RBD

**Figure 5:**
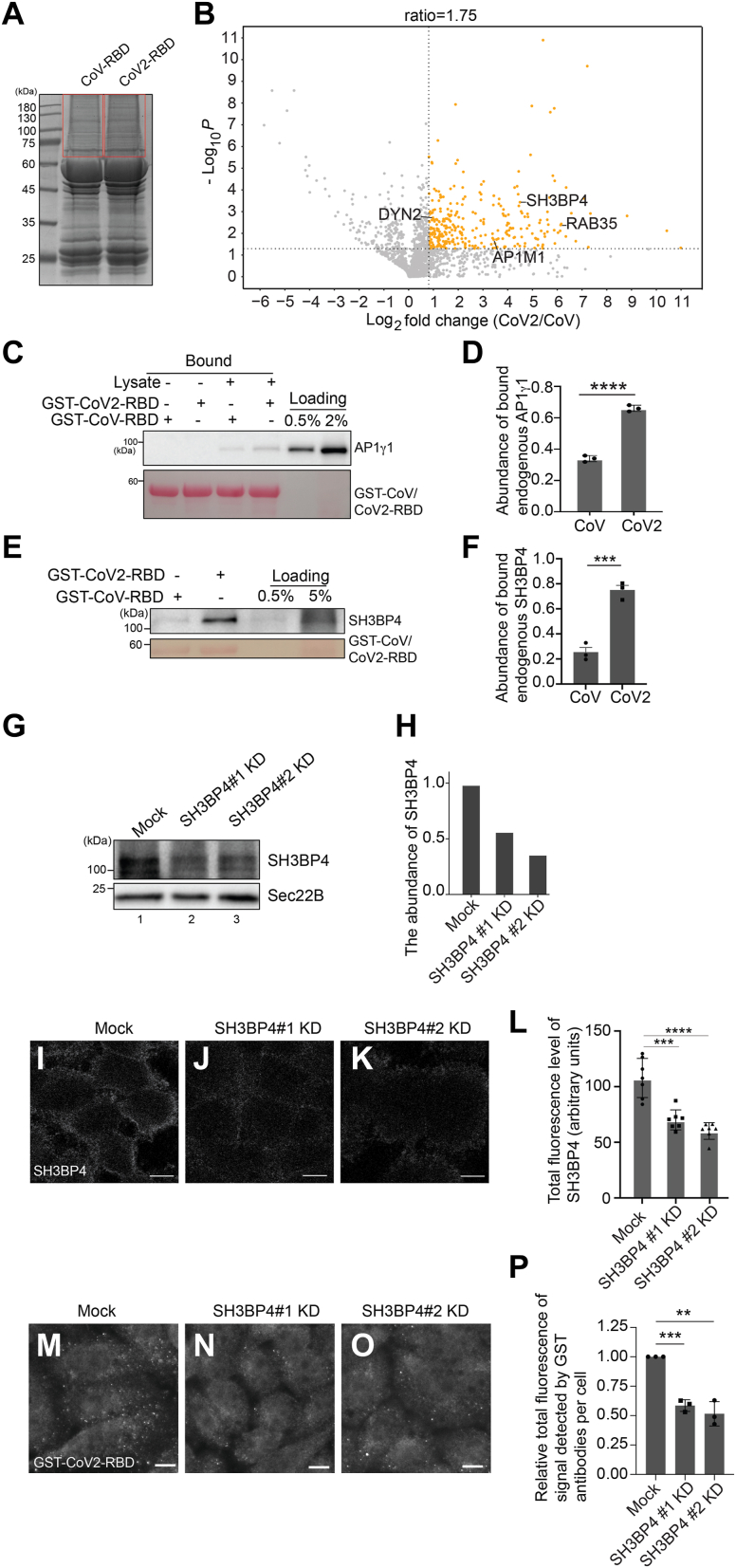
**Identification of host cell factors that preferentially bind to CoV2-RBD rather than CoV-RBD in HeLa cells.***A*, *C*, and *E*, purified GST-CoV-RBD or GST-CoV2-RBD was incubated with lysates from HeLa cells. After incubation, the bound proteins were analyzed by SDS-PAGE and Coomassie blue staining (*A*) or immunoblotting (*C* and *E*). *B*, volcano plot of mass spectrometry analysis of GST pull-down in (*A*). In-gel trypsin, digestion was performed based on the three biological repeats in (*A*) and the digested proteins were analyzed by label-free mass spectrometry. The mean log_2_ fold changes of the abundance of the identified proteins in the GST-CoV2-RBD group over that in the GST-CoV-RBD group were calculated and plotted against the minus log_10_*p* value. The *p* value was calculated by unpaired two-tail *t* test. *D* and *F*, relative abundances of AP1γ1 (*D*) and SH3BP4 (*F*) that bound to GST-CoV2-RBD or GST-CoV-RBD were quantified (n = 3, mean ± S.D.). In each biological repeat, the abundance of AP1γ1 or SH3BP4 that bound to GST-CoV2-RBD or GST-CoV-RBD was normalized to the corresponding bait protein, and this value was then normalized to the sum of abundance of bound proteins in all experimental groups. *G*, the levels of SH3BP4 and SEC22B in cell lysates from HeLa cells transfected with NC siRNA or with siRNAs against SH3BP4 were analyzed by immunoblotting with anti-SH3BP4 and anit-SEC22B antibodies. *H*, the levels of SH3BP4 and SEC22B in cell lysates from HeLa cells transfected with NC siRNA or siRNA against SH3BP4 were quantified. In each group, the level of SH3BP4 was normalized to the corresponding level of SEC22B, and this value was then normalized to the value of the Mock group. *I*–*K*, HeLa cells were transfected with NC siRNA (*B*) or siRNAs against SH3BP4 (*C* and *D*). Seventy two hours after transfection, SH3BP4 was detected by immunofluorescence. *L*, quantification of the total fluorescent signal of SH3BP4 in each cell (mean ± S.D., seven fields were quantified in each experimental group, over 30 cells in each fields). The scale bar represents 10 μm. *M*–*O*, HeLa cells were transfected with NC siRNA (*M*) or siRNAs against SH3BP4 (*N* and *O*). Forty eight hours after transfection, 5 ng/μl purified GST-CoV2-RBD was incubated with HeLa cells at 37 °C for 30 min. The presence of the purified protein was detected by immunofluorescence. The scale bar represents 10 μm. *P*, quantification of the total fluorescence of the signal in each cell (n = 3, mean ± S.D., over 100 cells were quantified in each experimental group). The level of the total fluorescence in each group was normalized to the level in Mock group. ∗∗*p* < 0.01; ∗∗∗*p* < 0.001; ∗∗∗∗*p* < 0.0001. AP1, adaptor protein complex.

The internalization analysis indicates that host factors but not ACE2 may preferentially interact with CoV2-RBD rather than CoV-RBD to induce internalization of CoV2-RBD in HeLa cells. We performed GST pull-down and label-free quantitative mass spectrometry to identify these factors. HeLa cell lysates were incubated with glutathione agarose beads bearing purified GST-CoV-RBD or GST-CoV2-RBD. The bound proteins were then analyzed by SDS-PAGE and Coomassie blue staining (Fig. 5*A*). The proteins migrated at the position above GST fusion proteins (highlighted in the red box in Fig. 5*A*) were then in-gel digested with trypsin and analyzed by label-free quantitative mass spectrometry based on three biological repeats. A total of 1728 proteins were identified and quantified, all of which had one or more unique peptides with a false discovery rate (FDR) of <0.01 and were successfully identified in all three biological repeats of the CoV2 group or the CoV group (Table S1, sheet 1). The fold changes of the identified proteins in the CoV2 group compared with the CoV group were quantified. Based on protein abundance, a *p* value was calculated. Minus log_10_ *p* value was plotted against the mean log_2_ fold changes (Fig. 5*B*). Proteins with a fold change of >1.75 and a *p* value of <0.05 were considered as significant hits. Through this approach, 316 proteins were identified as having more than 1.75-fold enrichment in the CoV2 group over the CoV group (*p* < 0.05) (Fig. 5*B*, highlighted in orange color) (Table S1, sheet 2). As HeLa cells express a low level of ACE2 (Fig. 4*L*), we did not identify ACE2 in all of the experimental groups (Table S1, sheet 1).

Among the 316 identified proteins, 39 proteins are predicted by UniProt to be located at the plasma membrane and four proteins, SH3BP4, dynamin-2, AP-1 complex subunit μ-1 (AP1M1), and Ras-related protein Rab-35 were localized to clathrin-coated vesicles (Fig. 5*B*, Table S1, sheet 3–4). SH3BP4 regulates the endocytosis of transferrin receptor (35). AP1M1 is a subunit of clathrin-associated adaptor protein complex 1 (AP1) that plays a role in protein sorting. We then focused our analysis on SH3BP4 and AP1M1. Unfortunately, we were unable to find commercially available antibodies against AP1M1 that can be used for immunoblotting. We therefore turned our attention to another subunit of the AP-1 complex, AP1γ1, and tested its interaction with SARS-CoV-2 RBD using commercial AP1γ1 antibodies. The Western blot analysis revealed that AP1γ1 binds more efficiently with GST-CoV2-RBD than GST-CoV-RBD (Fig. 5*C* and quantified in Fig. 5*D*). This finding is consistent with our mass spectrometry results. Western blot analysis confirmed that SH3BP4 showed a higher binding efficiency with GST-CoV2-RBD than with GST-CoV-RBD (Fig. 5*E* and quantification in Fig. 5*F*). Knockdown of SH3BP4 using two different siRNAs (Fig. 5, *G*–*L*) partially blocked the internalization of GST-CoV2-RBD in HeLa cells (Fig. 5, *M*–*O*, and quantification in Fig. 5*P*). These analyses indicate that SH3BP4 preferentially interacts with GST-CoV2-RBD rather than GST-CoV-RBD and is important for internalization of CoV2-RBD in HeLa cells.

**Figure 6:**
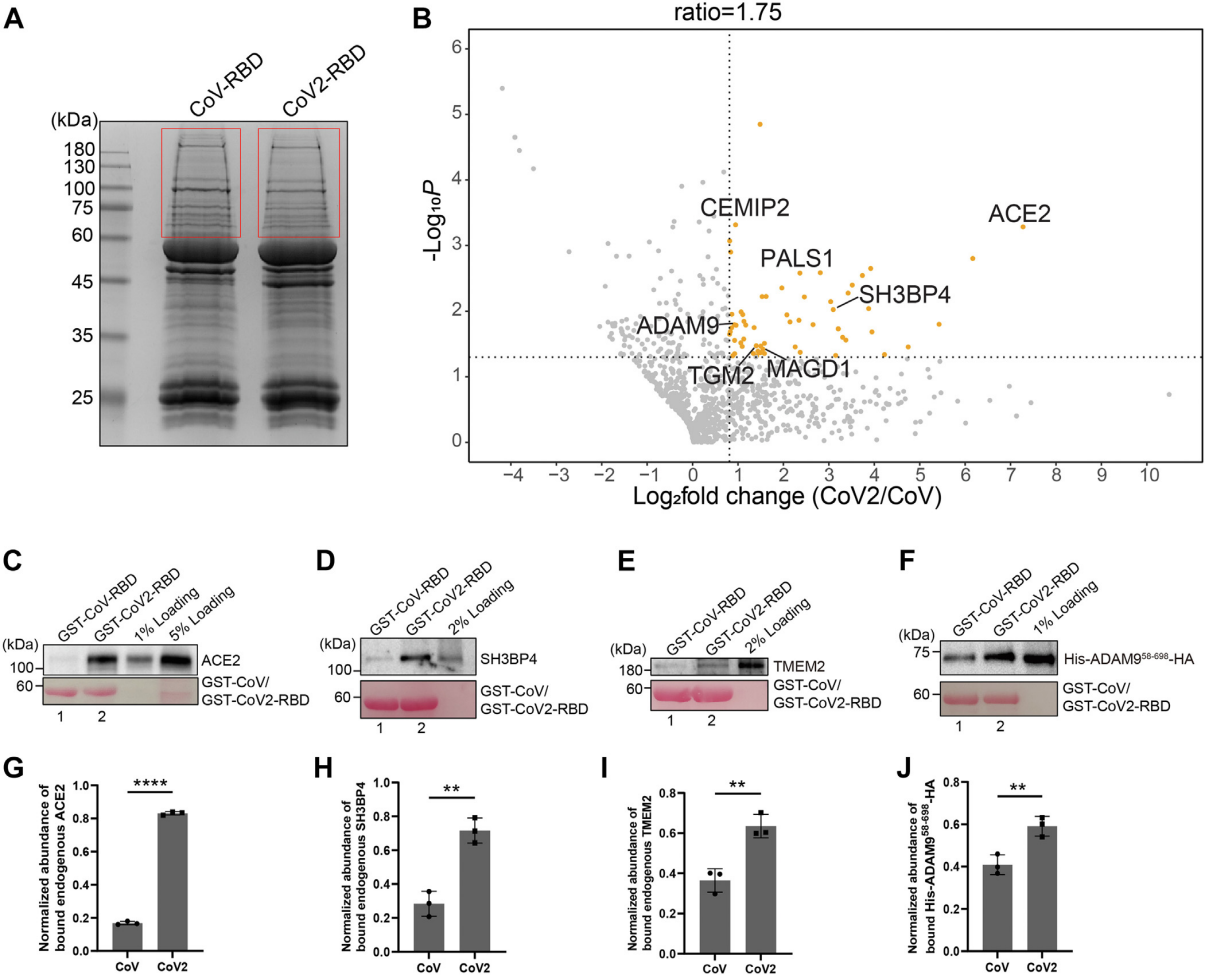
**Identification of host cell factors that preferentially bind to CoV2-RBD rather than CoV-RBD in Calu3 cells.***A* and *C*–*F*, purified GST-CoV-RBD or GST-CoV2-RBD was incubated with lysates from Calu-3 cells (*A* and *C*–*E*) or purified His- and HA-tagged extracellular domain of ADAM9 (*F*). After incubation, the bound proteins were analyzed by SDS-PAGE and Coomassie blue staining (*A*) or immunoblotting (*C*–*F*). *B*, volcano plot of mass spectrometry analysis of GST pull-down in (*A*). In-gel trypsin digestion was performed based on the three biological repeats in (*A*) and the digested proteins were analyzed by label-free mass spectrometry. The mean log_2_ fold changes in the identified proteins in the GST-CoV2-RBD group over the GST-CoV-RBD group were calculated and plotted against the minus log_10_*p* value. The *p* value was calculated by unpaired two-tail *t* test. *G*–*J*, relative abundances of ACE2 (*G*), SH3BP4 (*H*), TMEM2 (*I*), or His-ADAM9^58-698^-HA (*J*) that bound to GST-CoV2-RBD or GST-CoV-RBD were quantified (n = 3, mean ± S.D.). ∗∗*p* < 0.01; ∗∗∗∗*p* < 0.0001. In each biological repeat, the abundance of bound protein was normalized to the corresponding bait protein, and this value was then normalized to the sum of the normalized abundance of bound protein in all experimental groups. ACE2, angiotensin-converting enzyme 2.

The GST pull-down approach provides a convenient way to reveal novel binding partners of the receptor-binding domain of spike proteins. We then performed GST pull-down using lung epithelial Calu-3 cells that highly express ACE2 to reveal additional host cell factors that preferentially bind to CoV2-RBD rather than CoV-RBD. Cell lysates from Calu-3 cells were incubated with glutathione agarose beads bearing purified GST-CoV-RBD or GST-CoV2-RBD. The bound proteins were then analyzed by SDS-PAGE and Coomassie blue staining (Fig. 6*A*). The proteins migrated above the position of GST fusion proteins (highlighted in red frame in Fig. 6*A*) were then in-gel digested with trypsin and analyzed by label-free quantitative mass spectrometry based on three biological repeats. A total of 1049 proteins were identified and quantified, all of which had one or more unique peptides with an FDR of <0.01 and were successfully identified in all three biological repeats of the CoV2 group or the CoV group (Table S2, sheet 1). Sixty one proteins were identified as having more than 1.75-fold enrichment in the CoV2 group over the CoV group (*p* < 0.05) (Fig. 6*B*, highlighted in orange color) (Table S2, sheet 2). Among the 61 proteins, seven proteins including ACE2 were predicted by UniProt to be located at the cell surface (Fig. 6*B*, Table S2, sheet 3). SH3BP4 was also identified as one of the hits (Fig. 6*B*). Immunoblot analysis confirmed that the abundance of endogenous ACE2 and SH3BP4 that bound to GST-CoV2-RBD was significantly higher than that bound to GST-CoV-RBD (Fig. 6, *C* and *D* and quantification in Fig. 6, *G* and *H*).

We hypothesize that these proteins are host factors that interact preferentially to the receptor-binding domain of CoV2 spike proteins during the viral entry process. One of the identified proteins, transmembrane protein 2 (TMEM2; gene symbol CEMIP2), is a type II transmembrane protein. TMEM2 plays a critical role in promoting integrin-mediated cell adhesion and migration (36). Consistent with the mass spectrometry analysis, immunoblotting analysis indicates that TMEM2 has a higher binding efficiency with GST-CoV2-RBD than with GST-CoV-RBD (Fig. 6*E* and quantification in Fig. 6*I*). Another identified protein, membrane-anchored protein, disintegrin and metalloproteinase domain-containing protein 9 (ADAM9), is also a zinc metalloproteinase similar to ACE2. ADAM9 is highly expressed in lung and has been shown to be a key driver of SARS-CoV-2 replication (37) but the underlying mechanism is unknown. We found that purified His- and HA-tagged ADAM9 preferentially interacts with GST-CoV2-RBD compared to GST-CoV2-RBD (Fig. 6*F* and quantification in 6*J*), suggesting that ADAM9 may be another metalloproteinase that interacts with the receptor binding domain of SARS-CoV-2 to promote viral entry. The remaining three proteins from the initial seven identified were not examined.

Taken together, these analyses identified novel cell-surface–located host factors that preferentially interact with GST-CoV2-RBD rather than GST-CoV-RBD. These identified host factors may promote viral attachment and other processes, such as internalization, that promote entry of SARS-CoV-2.

### SH3BP4 regulates the entry of lentivirus pseudotyped with the prototype, the Delta, or the Omicron BA.2 variant of S protein

**Figure 7:**
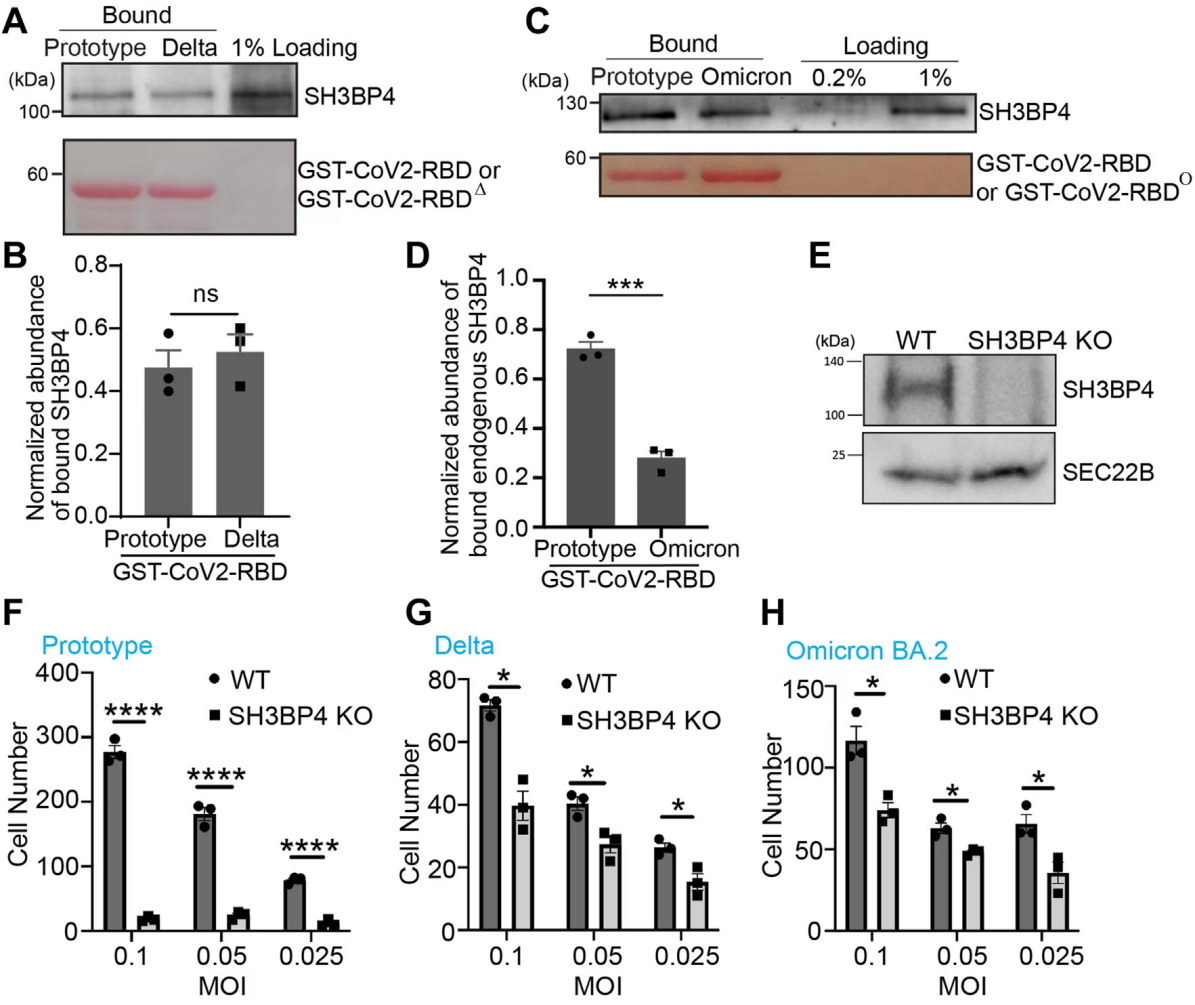
**SH3BP4 interacts with the Delta and Omicron variants of RBD and regulates entry of the lentivirus pseudotyped with the parental, Delta, or Omicron BA.2 variant of SARS-CoV-2 spike.***A* and *C*, purified GST-tagged prototype, Delta, and Omicron BA.1 variant of CoV2-RBD were incubated with lysates from Calu-3 cells. After incubation, the bound proteins were analyzed by immunoblot. *B* and *D*, relative abundances of SH3BP4 that bound to the indicated GST fusion proteins were quantified (n = 3, mean ± S.D.). In each biological repeat, the abundance of bound protein was normalized to the corresponding bait protein, and this value was then normalized to the sum of the normalized abundance of bound protein in all experimental groups. ∗∗∗*p* < 0.001; n.s., not significant. *E*, lysates from WT Vero E6 cells or from SH3BP4 KO Vero E6 cells were analyzed by immunoblot. *F*–*H*, the pseudovirus entry assay was performed using pseudotyped lentivirus carrying the prototype, Delta, and Omicron variant of BA.2 spike and WT and SH3BP4 KO Vero E6 cells (n = 3, mean ± S.D.). ∗*p* < 0.05; ∗∗∗∗*p* < 0.0001. MOI, multiplicity of infection.

Our previous results indicate that SH3BP4 preferentially interacts with CoV2-RBD rather than CoV-RBD and promotes the internalization of CoV2-RBD in HeLa cells (Fig. 5, *E*–*P*). GST pull-down analysis indicates that SH3BP4 also interacts with CoV2-RBD^Δ^ and the RBD from SARS-CoV-2 Omicron (CoV2-RBD^Ο^) (Fig. 7, *A*–*D*). The abundance of SH3BP4 that associated with CoV2-RBD^Δ^ was similar to that associated with CoV2-RBD (Fig. 7, *A* and *B*), whereas the abundance of SH3BP4 that associated with CoV2-RBD^Ο^ was lower than that associated with CoV2-RBD (Fig. 7, *C* and *D*).

We then performed pseudoviruses entry assays to test whether SH3BP4 is important for viral entry. WT Vero E6 cells or SH3BP4 KO Vero E6 cells (Fig. 7*E*) were incubated with lentiviruses pseudotyped with the prototype, the Delta or the Omicron BA2 variant of SARS-CoV-2 spike proteins (S^WT^, S^Δ^, or S^Ο^), and the efficiencies of viral entry were quantified. We found that KO of SH3BP4 significantly reduced the entry of lentiviruses pseudotyped with S^WT^, S^Δ^, or S^Ο^ (Fig. 7, *F*–*H*).

### Identification of additional host cell factors that preferentially bind to the Delta variant of GST-CoV2-RBD rather than the parental GST-CoV2-RBD

**Figure 8:**
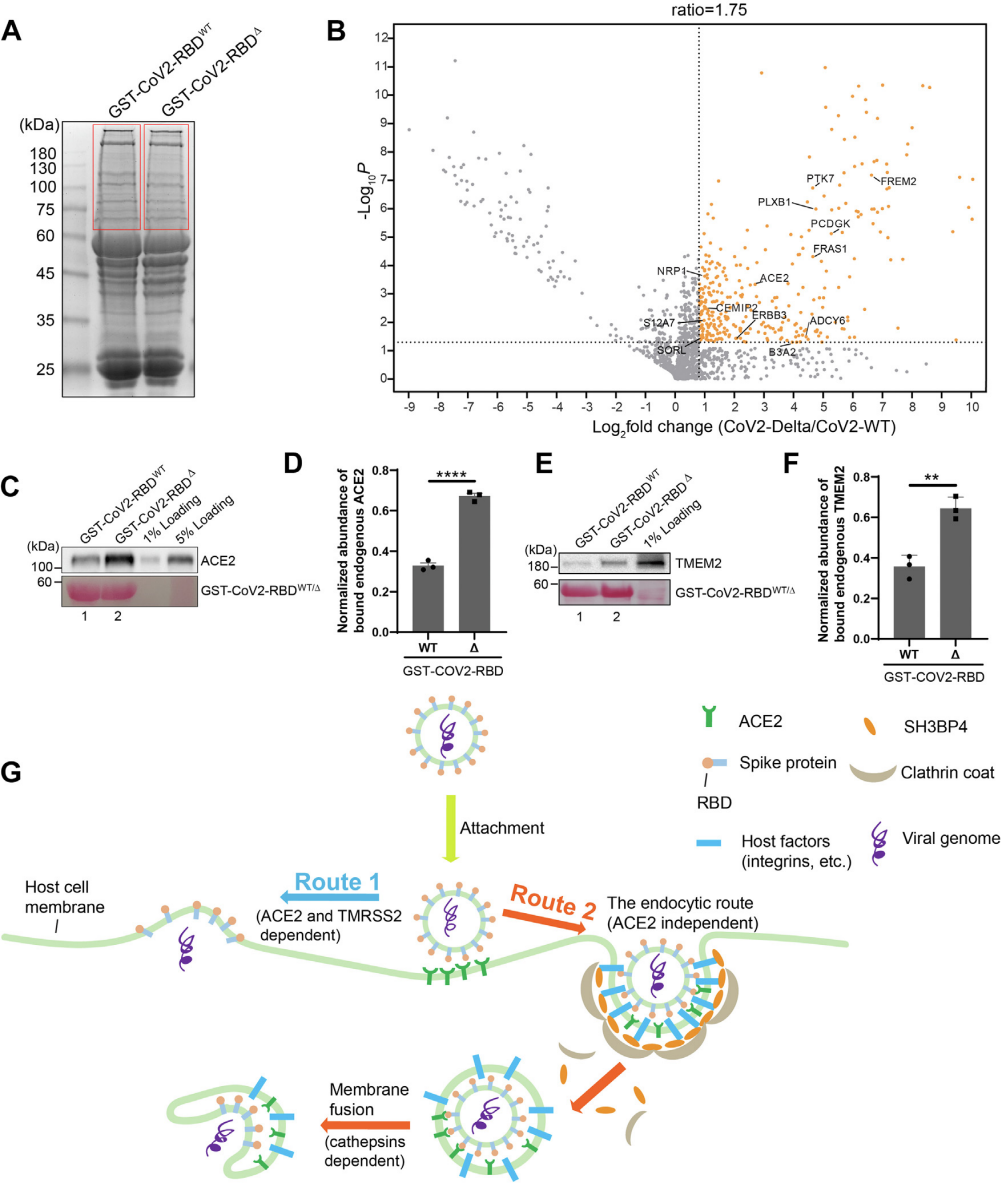
**Identification of additional host cell factors that preferentially bind to the Delta variant of CoV2-RBD rather than the WT CoV2-RBD in Calu3 cells.***A*, *C*, and *E*, purified GST-CoV2-RBD^WT^ or the Delta variant GST-CoV2-RBD^Δ^ was incubated with lysates from Calu-3 cells. After incubation, the bound proteins were analyzed by SDS-PAGE and Coomassie blue staining (*A*) or immunoblotting (*C* and *E*). *B*, volcano plot of mass spectrometry analysis of GST pull-down in (*A*). In-gel trypsin digestion was performed based on the three biological repeats in (*A*) and the digested proteins were analyzed by label-free mass spectrometry. The mean log_2_ fold changes in the abundance of the identified proteins in the GST-CoV2-RBD^Δ^ group over that in the GST-CoV2-RBD^WT^ group were calculated and plotted against the minus log_10_*p* value. The *p* value was calculated by unpaired two-tail *t* test. *D* and *F*, relative abundances of ACE2 (*D*) or TMEM2 (*F*) that bound to WT GST-CoV2-RBD or GST-CoV2-RBD^Δ^ were quantified (n = 3, mean ± S.D.). In each biological repeat, the abundance of bound protein was normalized to the corresponding bait protein, and this value was then normalized to the sum of the normalized abundance of bound protein in all experimental groups. ∗∗*p* < 0.01; ∗∗∗∗*p* < 0.0001. *G*, a model describes the entry of SARS-CoV2 *via* direct fusion with the plasma membrane (route 1) or *via* the endocytic route (route 2). ACE2, angiotensin-converting enzyme 2.

ACE2 was detected to preferentially bind to CoV2-RBD^Δ^ rather than the parental version (CoV2-RBD) (Fig. 2, *C* and *D*). Are there additional host factors that bind to CoV2-RBD^Δ^ rather than CoV2-RBD? To address this question, we performed GST pull-down and label-free quantitative mass spectrometry to reveal these host cell factors. Cell lysates from Calu-3 cells were incubated with glutathione agarose beads bearing purified GST-CoV2-RBD^Δ^ or GST-CoV2-RBD. The bound proteins were then analyzed by SDS-PAGE and Coomassie blue staining (Fig. 8*A*). The proteins highlighted in the red frame above the position of GST fusion proteins in SDS-PAGE gel (Fig. 8*A*) were then in-gel digested with trypsin and analyzed by label-free quantitative mass spectrometry based on three biological repeats. A total of 1592 proteins were identified and quantified, all of which had one or more unique peptides with an FDR of <0.01 and were successfully identified in all three biological repeats of the WT group or the Delta group (Table S3, sheet 1). Through this approach, 334 proteins were identified as having more than 1.75-fold enrichment in the Delta group over the WT group (*p* < 0.05) (Fig. 8*B*, highlighted in orange color) (Table S3, sheet 2). Among the 334 proteins, 60 proteins were predicted by UniProt to be located at the cell surface (Table S3, sheet 3). Thirteen proteins including ACE2 were predicted to be cell-surface–located transmembrane proteins (Fig. 8*B*, Table S3, sheet 4). Immunoblot analysis confirmed that the abundance of endogenous ACE2 that bound to GST-CoV2-RBD^Δ^ was significantly higher than that bound to GST-CoV2-RBD^WT^ (Fig. 8, *C* and *D*).

We hypothesize that these proteins are host factors that interact preferentially with the Delta variant of the receptor-binding domain of spike proteins during the viral entry process. We found that TMEM2 showed an around 2.2-fold enrichment in the Delta group over the WT group (Fig. 8*B*). TMEM2 also showed a preference for the CoV2-RBD over the CoV-RBD (Fig. 6, *B*, *E* and *I*). The increased interaction of TMEM2 with the Spike RBD during the virus mutation process suggests its potential significance in the context of viral infection. Therefore, we conducted GST pull-down assay to verify the interaction of TMEM2 with GST-CoV2-RBD^Δ^. As expected, TMEM2 showed higher binding efficiency with GST-CoV2-RBD^Δ^ than with GST-CoV2-RBD^WT^ (Fig. 8, *E* and *F*). The remaining 11 proteins from the initial 15 identified were not examined.

Taken together, these results identified ACE2, TMEM2, and other cell-surface–located host factors to preferentially interact with GST-CoV2-RBD^Δ^ compared to GST-CoV2-RBD. These identified host factors may promote entry of the Delta variant of SARS-CoV-2.

## Discussion

SARS-CoV-2 enters host cells either by direct fusion to the host cell membrane or by endocytosis. While the molecular mechanisms promoting membrane fusion are well understood, the means by which SARS-CoV-2 employs the endocytic pathway for its entry remain largely unknown. Our findings reveal that purified CoV2-RBD is internalized in HeLa cells in a clathrin- and integrin-dependent but ACE2-independent manner. In addition, we discovered that a host factor, SH3BP4, interacts with CoV2-RBD to regulate its endocytosis in HeLa cells and is important for the replication of lentivirus pseudotyped with the parental, Delta, and Omicron BA2 S proteins.

SH3BP4 is reported to modulate clathrin-mediated endocytosis of the transferrin receptor (35), Neuropilin-1 (NRP-1) and α5-integrin (38). Dephosphorylated SH3BP4 is recruited to clathrin-coated pits where it interacts with NRP1 and α5-integrin through its binding partner GIPC1 to facilitate internalization (38). Previous research has demonstrated that activation of integrin α5β1 promotes SARS-CoV-2 pseudovirus internalization independent of ACE2 (24). Similarly, NRP1 has been reported to regulate SARS-CoV-2 entry (24, 39, 40). Based on the published and our data, we hypothesize that SH3BP4 recognizes CoV2-RBD *via* host factors such as integrins or NRP1 to promote SARS-CoV-2 internalization and by a clathrin-dependent but ACE2-independent process (Fig. 8*G*, Route 2).

The integrin-binding motif (RGD) is located proximal to the ACE2-binding region in the S protein (21). Our investigations have revealed that the RGD motif is essential for the interactions between integrin β3 and CoV2-RBD (Fig. 3, *B*, *C*, *G* and *H*). Altering the RGD motif significantly impairs the uptake of GST-CoV2-RBD by HeLa cells (Fig. 4, *G*, *H* and *K*), emphasizing the critical role of this motif in CoV2 entry into host cells. Intriguingly, we found that mutating RGD motif leads to defects in CoV2-RBD-ACE2 interaction (Fig. 3, *D*, *E*, *I* and *J*), which suggests that RGD motif also plays a crucial role in the association between CoV2-RBD and ACE2, implicating a potential competitive dynamic between ACE2 and integrin for RBD binding. It is possible that integrin binding to the RGD motif enhances SARS-CoV-2 entry through the endocytic pathway while simultaneously blocking ACE2-binding site in the S protein thus hindering ACE2-initiated fusion of viral membrane to the host cell membrane. This hypothesis is supported by the findings that integrin inhibits the ACE2-S interaction (22, 41), and activation of integrin impairs SARS-CoV-2 pseudovirus particles entry in ACE2-expressing CHO-K1 cells (24).

Based on our study, we propose that the viral entry includes the following steps: (1) the ACE2-RBD interaction is key for the initial viral docking; (2) following this, the virus can either fuse directly with the host cell membrane to deliver its genome (Fig. 8*G*, route 1) or be internalized into the cell (Fig. 8*G*, route 2). We propose that route 1 is dependent on ACE2 and TMPRSS2, while route 2 does not rely on ACE2 but requires integrin and SH3BP4. Following the internalization step in the route 2, we speculate that the fusion of the viral and endosomal membranes is facilitated by cathepsins within an acidic environment (Fig. 8*G*, Route 2).

We have also identified ADAM9 and TMEM2 as interacting partners of CoV2-RBD. Like ACE2, ADAM9 is also a zinc metalloproteinase. Its metalloprotease domain mediates the cleavage of cell surface proteins, including epidermal growth factor, fibroblast growth factor receptor 2IIIb (FGFR2IIIb) (42), VCAM-1 (43), and Interleukin 11 (44). Recent studies have demonstrated that ADAM9 is a crucial factor in SARS-CoV-2 replication (37). Our findings show that purified His-tagged extracellular domain of ADAM9 directly interacts with GST-CoV2-RBD, exhibiting a stronger interaction than CoV-RBD. This suggests that SARS-CoV-2 may exploit ADAM9 to facilitate viral entry. TMEM2 is implicated in integrin-mediated cell adhesion and migration (36). Further investigation is needed to determine whether TMEM2 promotes viral entry.

ACE2 was initially identified as the receptor for SARS-CoV using an immunoprecipitation technique that involved the full S1 domain of S and metabolically labeled Vero-E6 cell lysates (45). Yet, our GST pull-down assay yielded only minimal binding between ACE2 and the SARS-CoV S RBD (Fig. 6*C*). There are several potential explanations for this weak interaction. One possibility is the difference in cell lines used for the assay: the previous study utilized Vero-E6 cells, while we used Calu-3 cells, and variations in cellular environment, posttranslational modifications, or cell-line–specific factors could affect the binding affinity of ACE2. Another possibility is the sensitivity of detection methods: the previous research utilized metabolic labeling with radioactive detection, which is more sensitive than our chemiluminescence method. Finally, the previous study utilizes immunoprecipitation to evaluate the interaction with the S1 subunit which differs from our GST pull-down approach to investigate the binding with the S-RBD.

The CoV2-RBD from the parental SARS-CoV-2 exhibits approximately 75% identity with CoV-RBD. We found that SH3BP4, ADAM9, and TMEM2 display enhanced binding to CoV2-RBD compared to CoV-RBD, suggesting that specific amino acid changes in CoV2-RBD enable SARS-CoV-2 to bind these host factors thereby potentially promoting infection. Most mutations in SARS-CoV-2 variants occur within the CoV2-RBD region, helping the virus evade immune surveillance. The impact of these mutations on the virus ability to bind additional surface-exposed host factors and facilitate entry remains unknown. We observed a higher binding efficiency between SH3BP4 and GST-CoV2-RBD^WT^ than GST-CoV2-RBD^Ο^ (Fig. 7, *C* and *D*), accompanied by a more substantial effect of SH3BP4 KO on the prototype virus entry than Omicron (Fig. 7, *F* and *H*). In contrast, our binding assays showed similar interactions between SH3BP4 and both GST-CoV2-RBD^WT^ and GST-CoV2-RBD^Δ^ (Fig. 7, *A* and *B*), but the knockout effect was indeed more pronounced for the prototype than for the Delta variant (Fig. 7, *F* and *G*). This could suggest that alternative cellular mechanisms might compensate for the absence of SH3BP4 in the entry of the Delta variant. Our mass spectrometry identified novel cell-surface–located transmembrane proteins that interacted preferentially with the RBD from SARS-CoV-2 Delta (RBD^Δ^) compared with RBD^WT^, suggesting that SARS-CoV-2 Delta may utilize these host factors to promote viral entry. The Omicron variant contains over 16 mutations in the RBD of its spike protein (46), which are correlated with its high capacity of transmission. The identify of new host factors that preferentially interact with the omicron variant of RBD should further help in understanding how Omicron achieves a high level of infection.

## Experimental procedures

### Constructs, reagents, cell culture, transfection, and immunofluorescence

HeLa cells and HEK293T cell lines were kindly provided by the University of California-Berkeley Cell Culture Facility. All cell lines were confirmed by short tandem repeat profiling and tested negative for *Mycoplasma* contamination. All cell lines were cultured in Dulbecco's Modified Eagle Medium (DMEM) containing 10% fetal bovine serum (FBS) and 1% penicillin streptomycin mix (Invitrogen).

For CRISPR-Cas9–mediated KO of SH3BP4 or ACE2, guide RNAs targeting SH3BP4 exon 2 (5′- GGGCGACCATCTCTACGTCT-3′) or ACE2 exon 1 (5′- AGAACAAGTCTTCGGCTTCG-3′) was designed using the CRISPR design tool (https://crisper.mit.edu) and cloned into pX458 (pSpCas9 BB-2A-GFP; Addgene, plasmid #48138). Multiple single cell–derived KO clones were isolated and screened for gene disruption by Western blotting. Cells were validated by immunoblotting and genomic PCR.

The cDNA encoding CoV-RBD, CoV2-RBD, human codon–optimized CoV2-RBD, human ACE2, and human integrin β3 were ordered from BGI. The plasmids encoding N-terminal GST-tagged CoV-RBD, N-terminal GST-tagged CoV2-RBD, C-terminal 3xFLAG-tagged human codon-optimized CoV2-RBD, C-terminal 3xHA-tagged ACE2^PD^, C-terminal GFP-tagged ACE2, and C-terminal 3xHA-tagged integrin β3 were generated by standard molecular cloning procedures. The plasmids encoding mutated versions of CoV2-RBD were generated by QuickChange II site-directed mutagenesis using plasmids encoding GST-tagged CoV2-RBD, 3xFLAG-tagged human codon optimized CoV2-RBD as templates.

siRNAs against CHC and SH3BP4 were purchased from Ribo-bio. The target sequence of the siRNA against CHC is TAATCCAATTCGAAGACCAAT (34). The target sequence of the two siRNAs against SH3BP4 is CCACGAATAGCACTGGCAA and GGAACTCAACACTGAGTGA, respectively. The commercial antibodies were rabbit anti-HA (Cell Signaling, catalog number 3724), rabbit anti-FLAG (Sigma-Aldrich, catalog number F7425), goat anti-GST (GE Healthcare, catalog number 27-4577), rabbit anti-ACE2 (Abcam, catalog number ADI-SPA-891-F), sheep anti-TGN46 (BIO-RAD, catalog number ab15348), mouse anti-β-action (Proteintech, catalog number 60008-1), mouse anti-SH3BP4 (Santa Cruz, catalog number sc-393730), TMEM2 (Sigma-Aldrich, catalog number HPA044889). Synthetic S1 peptides (GGNYNYLYRLFRK), S2 peptides (YFPLQSYGFQPTNGVGY), and ACE2 peptides (QAKTFLDKFNHEAEDLFYQSSL) were purchased from GenScript.

Transfection of siRNA or DNA constructs into HeLa cells or HEK293T cells and immunofluorescence were performed as described previously (47). Images were acquired with a Zeiss Axio Observer Z1 microscope system (Carl Zeiss) equipped with an ORCA Flash 4.0 camera (Hamamatsu) or Leica STED TCS SP5 II Confocal Laser Scanning Microscope (Leica). The total fluorescence of internalized GST-CoV-RBD, GST-CoV2-RBD, and mutated GST-CoV2-RBD labelled by GST antibody was calculated by Fiji.

### Protein purification, GST pull-down, internalization assay, and immunoprecipitation

Purification of GST-CoV-RBD, GST-CoV2-RBD, and mutated GST-CoV2-RBD was performed as described previously (48). GST pull-down assays were carried out with 10 μl of compact GSH beads bearing around 5 μg of GST-CoV-RBD, GST-CoV2-RBD, or mutated GST-CoV2-RBD. The beads were incubated with 200 μl of 0.5 mg/ml of cell lysates from HEK293T cells transfected with ACE2^PD^-HA or integrin β3-HA or 2 ml of 0.5 mg/ml of cell lysates from Calu-3 cells in HKT buffer (100 mM KCl, 20 mM Hepes, pH 7.2, 0.5% Triton X-100) at pH 6.0 or 7.2 with mixing at 4 °C overnight. After incubation, the beads were washed three times with 500 μl of HKT buffer and twice with 500 μl of HK buffer (100 mM KCl, 20 mM Hepes, pH 7.2), and the bound material was analyzed by Coomassie Blue (Bio-Safe Coomassie-G250) staining and immunoblotting.

Internalization assay was performed by incubating HeLa cells or Vero E6 cells with 5 ng/μl purified GST-tagged CoV-RBD, CoV2-RBD, or mutated CoV2-RBD at 37 °C for 30 min. After incubation, the presence of the purified protein was detected by immunofluorescence.

Immunoprecipitation was performed by incubating 200 μl of 0.5 mg/ml cell lysates from HEK293T cells cotransfected with human codon-optimized CoV2-RBD-FLAG and ACE2^PD^-HA or integrin β3-HA in HKT buffer with 10 μl of compact anti-HA agarose affinity beads with mixing at 4 °C overnight. After incubation, the beads were washed four times with 1 ml of HK buffer, and the bound material was analyzed by immunoblotting.

### Mass spectrometry–based label-free quantitative proteomics

#### Sample preparation

The host cell factors that bind to CoV-RBD, CoV2-RBD, or the Delta variant of CoV2-RBD were determined by mass spectrometry–based label-free quantitative proteomics approach. GST pull-down assay was performed using HeLa or Calu-3 cells and GST-tagged CoV-RBD, CoV2-RBD, or the Delta variant of CoV2-RBD, and the bound proteins were analyzed by Coomassie Blue staining.

To identify the pulled-down proteins by mass spectrometry–based proteomics approach, the protein gel was subjected to in-gel digestion with a typical procedure as described previously (49). Briefly, the protein gel was cut into small fragments and dehydrated with acetonitrile for 15 min. Subsequently, disulfide bonds of proteins were reduced with 10 mM TCEP at 55 °C for 45 min and then alkylated with 55 mM iodoacetamide at room temperature in dark for 45 min. The proteins were then digested with sequencing-grade modified trypsin (Promega) at 37 °C overnight. The resulting peptides were extracted from the protein gel with 60% acetonitrile containing 5% formic acid and the extract was dried with a vacuum concentrator. The dried sample was then reconstituted in 0.1% TFA and desalted with a C18 spin column (Thermo Fisher Scientific) according to the procedure suggested by the manufacturer. The desalted sample was dried with a vacuum concentrator and stored under −20 °C until the LC/MS analysis.

#### LC/MS analysis

LC/MS analysis was performed using a Bruker Daltonics nanoElute LC coupled to a Bruker Daltonics timsTOF Pro trapped ion mobility (TIMS) quadrupole time-of-flight mass spectrometer with a CaptiveSpray nanoelectrospray ion source. The dried desalted samples were reconstituted in 10 μl of 0.1% formic acid in water, and an aliquot of 1 μl of which was injected for LC/MS analysis. LC separation was carried out with an ionopticks C18 Aurora Series emitter column with CaptiveSpray insert (75 μm ID × 25 cm, 1.6 μm). Mobile phase A was 98% milliQ water/2% acetonitrile (v/v) with 0.1% (v/v) formic acid, and mobile phase B was 100% acetonitrile with 0.1% formic acid (v/v). Peptides were eluted at a flow rate of 0.3 μl/min with a solvent gradient: 0 to 0.5 min: 2 to 5% B, 0.5 to 27 min: 5 to 30% B, 27 to 27.5 min: 30 to 95% B, 27.5 to 28 min: 95% B, 28 to 28.1 min: 95-2% B, 28.1 to 30 min: 2% B. The column temperature was set at 50 °C.

The mass spectrometer was operated in positive ion mode. Mass calibration was performed with sodium formate and TIMS calibration was carried out with the Agilent ESI tuning mix [*m/z*,1/K_0_: (622.0289,0.9848 V⋅s/cm^2^), (922.0097, 1.1895 V⋅s/cm^2^), (1221,9906, 1.3820 V⋅s/cm^2^)]. During data acquisition, the mass spectrometer was operated in data-dependent acquisition-Parallel Accumulation-Serial Fragmentation mode with a total cycling time of 0.53 s including 1 full MS scan and 4 PASEF MS/MS scans. MS and MSMS spectra were acquired with an *m/z* range of 100 to 1700 and TIMS scan range of 0.85 to 1.3 V⋅s/cm^2^. The TIMS ramp time was set at 100 ms.

#### Data analysis

Protein identification and label-free quantification were executed using the PEAKS Studio version Xpro (Bioinformatics Solutions Inc). The database search parameters for protein identification were as follows: taxonomy: *Homo sapiens* (UniProt); fixed modification: cysteine carbamidomethylation (+57.021 Da); variable modification: methionine oxidation (+15.995 Da) and acetylation (+42.011) at the Protein N terminus; protease: trypsin; missed cleavage allowed: 2; mass tolerance: 15 ppm for precursor ion and 0.06 Da for fragment ion. Peptide and protein matches with FDR <1% and protein matches with at least one unique peptide were considered to be significant.

For quantitative analysis, protein abundance was determined based on the peak areas of all identified unique peptides. Each biological repeat contains two experimental groups for comparison. Only proteins fulfilling the significant matching criteria (FDR < 1% and number of unique peptides ≥1) and successfully identified in all three biological replicates of at least one of the two experimental groups will be selected for quantitative analysis. The protein abundance was assigned to 20 if not found. For each biological repeat, the abundance of each protein (referred to as a) in a sample was normalized to the average value of protein abundance of all identified proteins in each sample (referred to as a-average). This normalization (a/a-average) is to adjust the total abundance of bound proteins between the two experimental groups in each biological repeat to be equal. As the replicated experiments were performed at different periods, the normalized abundance of a specific protein that bound to a specific bait protein may be different among different replicates due to slight variations of experimental conditions. To avoid this variation, we performed a second normalization. Here the value of a/a-average of a specific identified protein in one sample was normalized again to the sum of the value of a/a-average of this identified protein in two experimental groups of each biological repeat. The *p*-value was calculated using the normalized abundance of each identified protein using a two-tailed student *t* test.

### Pseudovirus production

HEK-293FT cells were transfected with pseudotyping vectors from InvivoGen (Original D614, Delta or Omicron BA2 spike), combining with pLenti-CMV-GFP-Puro (#17448 Addgene) and psPAX2 (#12260 Addgene) for lentivirus packaging. The supernatant of cell culture was harvested at 96 h post-infection, followed by filtration with 0.45 μm filter and precipitation with PEG-it virus precipitation solution (5x) (System Biosciences) in 4 °C for 12 h. The supernatant was discarded after centrifugation at maximum speed for 30 min, and the precipitated virus was eluted with pre-cold PBS for following usage or stock in −80 °C.

### Virus titrations

The virus stock titeres were determined by preparing 10-fold serial dilutions in Opti-MEM (Thermo Fisher Scientific). One hundred μl of each dilution was added to monolayers of HEK-293T cells that stably expressing human ACE2 (293T-hACE2). After infection at 37 °C for 3 h, the virus solution was discarded and incubated with DMEM + 10% FBS for 96 h. The signal of GFP was detected using 500 to 550 nm emission filters with Cytation 7 Cell Imaging Multimode Reader (BioTek). The number of GFP-positive infected cells were quantified using BioTek Gen5 software (version 3.12, BioTek, https://www.agilent.com/en/product/cell-analysis/cell-imaging-microscopy/cell-imaging-microscopy-software/biotek-gen5-software-for-imaging-microscopy-1623226) and indicating the titration of packaged viruses.

### Pseudovirus entry assay

Vero and Vero-SH3BP4 KO cells were pre-seeded for 12 h prior to transduction and the culture medium was discarded before infection. The indicated packaged virus diluted with Opti-MEM (Thermo Fisher Scientific) were transduced to monolayers of Vero or Vero-SH3BP4 KO cells at MOI = 0.1, 0.05, and 0.025, respectively. After incubation at 37 °C for 3 h, the virus solution was discarded and incubated with DMEM + 10% FBS for 96 h. The signal of GFP was detected using 500 to 550 nm emission filters with Cytation 7 Cell Imaging Multimode Reader (BioTek). The number of GFP-positive infected cells were quantified using BioTek Gen5 software (version 3.12, BioTek). All the assays were performed in triplicate.

## Data availability

All data associated with this study are available within the article and its supporting information file. Raw data are available upon request from the corresponding authors.

## Supporting information

This article contains supporting information.

## Conflict of interest

The authors declare that they have no conflicts of interest with the contents of this article.

## References

[1] Zhou P., Yang X.L., Wang X.G., Hu B., Zhang L., Zhang W. (2020). A pneumonia outbreak associated with a new coronavirus of probable bat origin. Nature.

[2] Walls A.C., Park Y.J., Tortorici M.A., Wall A., McGuire A.T., Veesler D. (2020). Structure, function, and Antigenicity of the SARS-CoV-2 spike Glycoprotein. Cell.

[3] Letko M., Marzi A., Munster V. (2020). Functional assessment of cell entry and receptor usage for SARS-CoV-2 and other lineage B betacoronaviruses. Nat. Microbiol..

[4] Hoffmann M., Kleine-Weber H., Schroeder S., Krüger N., Herrler T., Erichsen S. (2020). SARS-CoV-2 cell entry Depends on ACE2 and TMPRSS2 and is blocked by a Clinically proven protease inhibitor. Cell.

[5] Hartenian E., Nandakumar D., Lari A., Ly M., Tucker J.M., Glaunsinger B.A. (2020). The molecular virology of Coronaviruses. J. Biol. Chem..

[6] Jackson C.B., Farzan M., Chen B., Choe H. (2022). Mechanisms of SARS-CoV-2 entry into cells. Nat. Rev. Mol. Cell Biol..

[7] Wrapp D., Wang N., Corbett K.S., Goldsmith J.A., Hsieh C.L., Abiona O. (2020). Cryo-EM structure of the 2019-nCoV spike in the prefusion conformation. Science.

[8] Lan J., Ge J., Yu J., Shan S., Zhou H., Fan S. (2020). Structure of the SARS-CoV-2 spike receptor-binding domain bound to the ACE2 receptor. Nature.

[9] Gordon D.E., Jang G.M., Bouhaddou M., Xu J., Obernier K., White K.M. (2020). A SARS-CoV-2 protein interaction map reveals targets for drug repurposing. Nature.

[10] Stukalov A., Girault V., Grass V., Karayel O., Bergant V., Urban C. (2021). Multilevel proteomics reveals host perturbations by SARS-CoV-2 and SARS-CoV. Nature.

[11] Starr T.N., Greaney A.J., Hilton S.K., Ellis D., Crawford K.H.D., Dingens A.S. (2020). Deep mutational Scanning of SARS-CoV-2 receptor binding domain reveals Constraints on folding and ACE2 binding. Cell.

[12] Wang Q., Zhang Y., Wu L., Niu S., Song C., Zhang Z. (2020). Structural and functional basis of SARS-CoV-2 entry by using human ACE2. Cell.

[13] Saberiyan M., Karimi E., Khademi Z., Movahhed P., Safi A., Mehri-Ghahfarrokhi A. (2022). SARS-CoV-2: phenotype, genotype, and characterization of different variants. Cell. Mol. Biol. Lett..

[14] Han P., Li L., Liu S., Wang Q., Zhang D., Xu Z. (2022). Receptor binding and complex structures of human ACE2 to spike RBD from omicron and delta SARS-CoV-2. Cell.

[15] Wang Y., Liu C., Zhang C., Wang Y., Hong Q., Xu S. (2022). Structural basis for SARS-CoV-2 Delta variant recognition of ACE2 receptor and broadly neutralizing antibodies. Nat. Commun..

[16] Sinha S., Tam B., Wang S.M. (2021). RBD double mutations of SARS-CoV-2 Strains Increase transmissibility through enhanced interaction between RBD and ACE2 receptor. Viruses.

[17] Tian F., Tong B., Sun L., Shi S., Zheng B., Wang Z. (2021). N501Y mutation of spike protein in SARS-CoV-2 strengthens its binding to receptor ACE2. Elife.

[18] Barton M.I., MacGowan S.A., Kutuzov M.A., Dushek O., Barton G.J., van der Merwe P.A. (2021). Effects of common mutations in the SARS-CoV-2 Spike RBD and its ligand, the human ACE2 receptor on binding affinity and kinetics. Elife.

[19] Kim S., Liu Y., Lei Z., Dicker J., Cao Y., Zhang X.F., Im W. (2021). Differential interactions between human ACE2 and spike RBD of SARS-CoV-2 variants of concern. bioRxiv.

[20] Makowski L., Olson-Sidford W., W-Weisel J. (2021). Biological and clinical consequences of integrin binding via a Rogue RGD motif in the SARS CoV-2 spike protein. Viruses.

[21] Sigrist C.J., Bridge A., Le Mercier P. (2020). A potential role for integrins in host cell entry by SARS-CoV-2. Antiviral Res.

[22] Gao S., Lu Y., Luan J., Zhang L. (2021). Low incidence rate of diarrhoea in COVID-19 patients is due to integrin. J. Infect..

[23] Beddingfield B.J., Iwanaga N., Chapagain P.P., Zheng W., Roy C.J., Hu T.Y. (2021). The integrin binding peptide, ATN-161, as a novel Therapy for SARS-CoV-2 infection. JACC Basic Transl Sci..

[24] Liu J., Lu F., Chen Y., Plow E., Qin J. (2022). Integrin mediates cell entry of the SARS-CoV-2 virus independent of cellular receptor ACE2. J. Biol. Chem..

[25] Ludwig B.S., Kessler H., Kossatz S., Reuning U. (2021). RGD-binding integrins revisited: how recently discovered functions and novel synthetic ligands (Re-)Shape an ever-evolving field. Cancers (Basel).

[26] Liu Z., Wang F., Chen X. (2008). Integrin alpha(v)beta(3)-Targeted Cancer Therapy. Drug Dev. Res..

[27] Ria R., Vacca A., Ribatti D., Di Raimondo F., Merchionne F., Dammacco F. (2002). Alpha(v)beta(3) integrin engagement enhances cell invasiveness in human multiple myeloma. Haematologica.

[28] Bledzka K., Smyth S.S., Plow E.F. (2013). Integrin αIIbβ3: from discovery to efficacious therapeutic target. Circ. Res..

[29] Ogawa J., Zhu W., Tonnu N., Singer O., Hunter T., Ryan A.L., Pao G.M. (2020). The D614G mutation in the SARS-CoV2 Spike protein increases infectivity in an ACE2 receptor dependent manner. bioRxiv.

[30] Zech F., Schniertshauer D., Jung C., Herrmann A., Cordsmeier A., Xie Q. (2021). Spike residue 403 affects binding of coronavirus spikes to human ACE2. Nat. Commun..

[31] Bayati A., Kumar R., Francis V., McPherson P.S. (2021). SARS-CoV-2 infects cells after viral entry via clathrin-mediated endocytosis. J. Biol. Chem..

[32] Fu Y., Xiong S. (2021). Tagged extracellular vesicles with the RBD of the viral spike protein for delivery of antiviral agents against SARS-COV-2 infection. J. Control Release.

[33] Prabhakara C., Godbole R., Sil P., Jahnavi S., Gulzar S.E.J., van Zanten T.S. (2021). Strategies to target SARS-CoV-2 entry and infection using dual mechanisms of inhibition by acidification inhibitors. PLoS Pathog..

[34] Ma T., Li B., Wang R., Lau P.K., Huang Y., Jiang L. (2018). A mechanism for differential sorting of the planar cell polarity proteins Frizzled6 and Vangl2 at the trans-Golgi network. J. Biol. Chem..

[35] Tosoni D., Puri C., Confalonieri S., Salcini A.E., De Camilli P., Tacchetti C. (2005). TTP specifically regulates the internalization of the transferrin receptor. Cell.

[36] Irie F., Tobisawa Y., Murao A., Yamamoto H., Ohyama C., Yamaguchi Y. (2021). The cell surface hyaluronidase TMEM2 regulates cell adhesion and migration via degradation of hyaluronan at focal adhesion sites. J. Biol. Chem..

[37] Carapito R., Li R., Helms J., Carapito C., Gujja S., Rolli V. (2022). Identification of driver genes for critical forms of COVID-19 in a deeply phenotyped young patient cohort. Sci. Transl. Med..

[38] Burckhardt C.J., Minna J.D., Danuser G. (2021). SH3BP4 promotes neuropilin-1 and alpha5-integrin endocytosis and is inhibited by Akt. Dev. Cell.

[39] Cantuti-Castelvetri L., Ojha R., Pedro L.D., Djannatian M., Franz J., Kuivanen S. (2020). Neuropilin-1 facilitates SARS-CoV-2 cell entry and infectivity. Science.

[40] Daly J.L., Simonetti B., Klein K., Chen K.E., Williamson M.K., Antón-Plágaro C. (2020). Neuropilin-1 is a host factor for SARS-CoV-2 infection. Science.

[41] Luan J., Lu Y., Gao S., Zhang L. (2020). A potential inhibitory role for integrin in the receptor targeting of SARS-CoV-2. J. Infect..

[42] Peduto L., Reuter V.E., Shaffer D.R., Scher H.I., Blobel C.P. (2005). Critical function for ADAM9 in mouse prostate cancer. Cancer Res..

[43] Guaiquil V., Swendeman S., Yoshida T., Chavala S., Campochiaro P.A., Blobel C.P. (2009). ADAM9 is involved in pathological retinal neovascularization. Mol. Cell. Biol..

[44] Sammel M., Peters F., Lokau J., Scharfenberg F., Werny L., Linder S. (2019). Differences in Shedding of the Interleukin-11 receptor by the proteases ADAM9, ADAM10, ADAM17, meprin alpha, meprin beta and MT1-MMP. Int. J. Mol. Sci..

[45] Li W., Moore M.J., Vasilieva N., Sui J., Wong S.K., Berne M.A. (2003). Angiotensin-converting enzyme 2 is a functional receptor for the SARS coronavirus. Nature.

[46] Wang Q., Guo Y., Iketani S., Nair M.S., Li Z., Mohri H. (2022). Antibody evasion by SARS-CoV-2 Omicron subvariants BA.2.12.1, BA.4 and BA.5. Nature.

[47] Tang X., Zhang L., Ma T., Wang M., Li B., Jiang L. (2020). Molecular mechanisms that regulate export of the planar cell-polarity protein Frizzled-6 out of the endoplasmic reticulum. J. Biol. Chem..

[48] Guo Y., Zanetti G., Schekman R. (2013). A novel GTP-binding protein-adaptor protein complex responsible for export of Vangl2 from the trans Golgi network. Elife.

[49] Pisamai S., Roytrakul S., Phaonakrop N., Jaresitthikunchai J., Suriyaphol G. (2018). Proteomic analysis of canine oral tumor tissues using MALDI-TOF mass spectrometry and in-gel digestion coupled with mass spectrometry (GeLC MS/MS) approaches. PLoS One.

