# Supplementary material for: Identifications of novel host cell factors that interact with the receptor-binding domain of the SARS-CoV-2 spike protein
Source: J Biol Chem. 2024 May 21;300(6):107390. doi: 10.1016/j.jbc.2024.107390 (PMC11237930; doi:10.1016/j.jbc.2024.107390)
Supplement: Supporting Information [file mmc1.pdf]

Supplemental Information

Identifications of novel host cell factors that interact with the receptor binding domain of the SARS-CoV-2 spike protein

Xiao Tang[1,2#], Yang Liu[2, #], Jinhui Wang[2, #], Teng Long[3, 4, #], Bobo Wing Yee Mok[3, 4], Yan Huang[2], Ziqing Peng[2], Qinyu Jia[5], Chengxi Liu[5], Pui-Kin So[5], Sirius Pui-Kam Tse[5], Cheuk Hei NG[2], Shiyi Liu[6], Fei Sun[7], Shaojun Tang[6], Zhong-ping Yao[5], Honglin Chen[3,4], Yusong Guo[2,8,*].

1. Anhui Provincial Key Laboratory of Molecular Enzymology and Mechanism of Major Diseases, Auhui Provincial Engineering Research Centre for Molecular Detection and Diagnostics, College of Life Sciences, Anhui Normal University, Wuhu, China;
2. Division of Life Science and State Key Laboratory of Molecular Neuroscience, The Hong Kong University of Science and Technology, Hong Kong;
3. Department of Microbiology, The University of Hong Kong, Hong Kong SAR, China;
4. Centre for Virology, Vaccinology and Therapeutics Limited, The University of Hong Kong, Pokfulam, Hong Kong SAR, China;
5. State Key Laboratory of Chemical Biology and Drug Discovery, Research Institute for Future Food and Department of Applied Biology and Chemical Technology, The Hong Kong Polytechnic University, Hung Hom, Kowloon, Hong Kong Special Administrative Region, China;
6. Thrust of Bioscience and Biomedical Engineering, Hong Kong University of Science and Technology (Guangzhou), China;
7. Department of Chemical and Biological Engineering, Hong Kong University of Science and Technology, Hong Kong SAR, China;
8. Hong Kong University of Science and Technology, Shenzhen Research Institute, Shenzhen, China.

#, These authors contribute equally.
*, Corresponding author: guoyusong@ust.hk

Figure S1

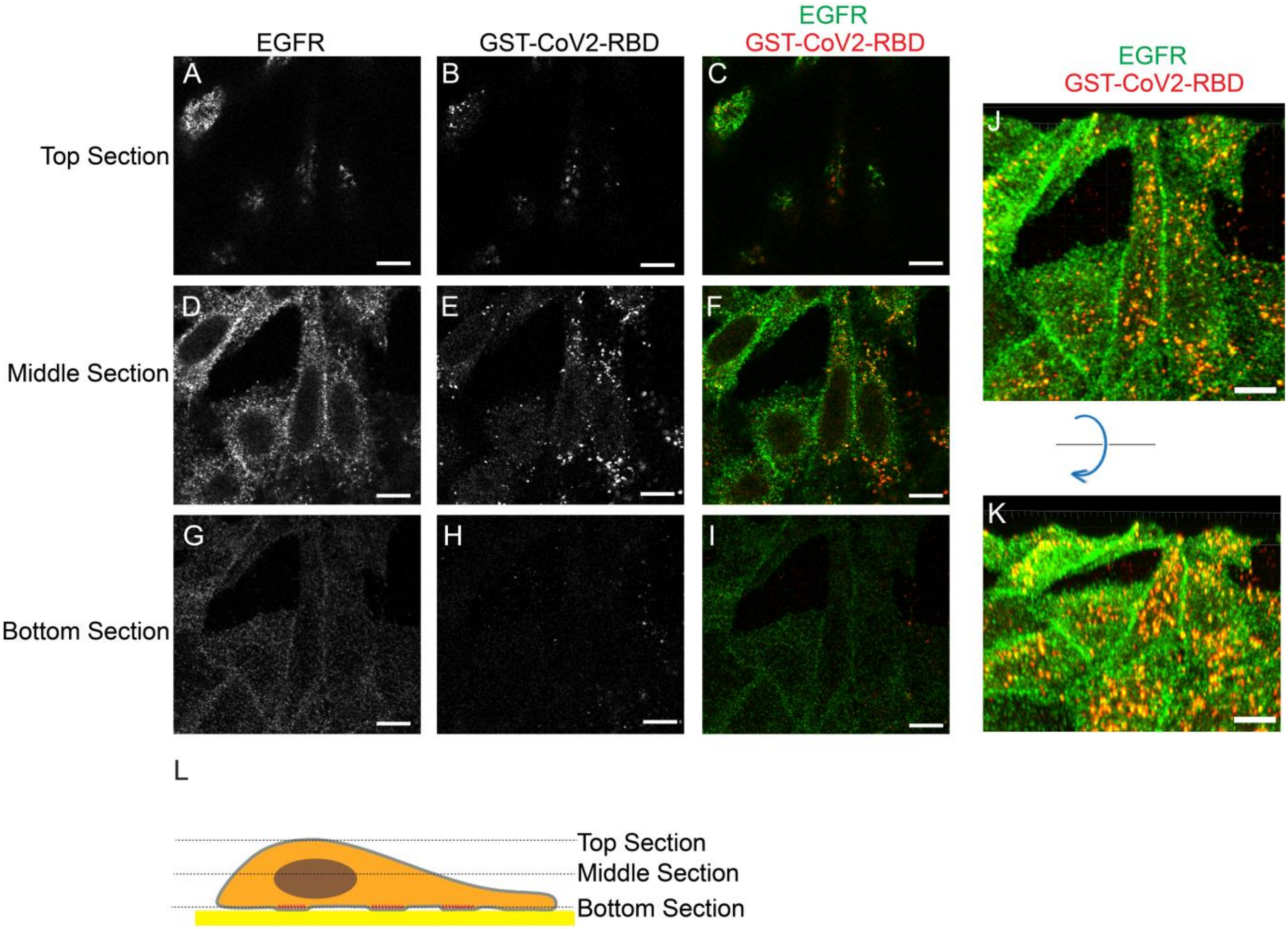

Figure S1. 3D image of GST-CoV2-RBD internalized into HeLa cells. A-K. 5 ng/μl purified GST-CoV2-RBD was incubated with HeLa cells at 37°C. 30 min after incubation, the presence of the purified protein was detected by immunofluorescence by anti-GST antibodies. The cell surface was detected by immunofluorescence by anti-EGFR antibodies. Three representative confocal optical sections, top (A-C), middle (D-F), and bottom (G-I), were taken along the z axis of HeLa cells. *Scale bar,* 10 μm. **J-K.** The localizations of EGFR and GST-CoV2-RBD in 3D. *Scale bar,* 10 μm. **L.** Diagram shows three sections of HeLa cells.
